# A kinetic investigation of interacting, stimulated T cells identifies conditions for rapid functional enhancement, minimal phenotype differentiation, and improved adoptive cell transfer tumor eradication

**DOI:** 10.1371/journal.pone.0191634

**Published:** 2018-01-23

**Authors:** Jing Zhou, Michael T. Bethune, Natalia Malkova, Alexander M. Sutherland, Begonya Comin-Anduix, Yapeng Su, David Baltimore, Antoni Ribas, James R. Heath

**Affiliations:** 1 NanoSystems Biology Cancer Center, California Institute of Technology, Pasadena, California, United States of America; 2 Division of Chemistry and Chemical Engineering, California Institute of Technology, Pasadena, California, United States of America; 3 Division of Biology and Biological Engineering, California Institute of Technology, Pasadena, California, United States of America; 4 David Geffen School of Medicine, the Johnson Comprehensive Cancer Center, University of California, Los Angeles, California, United States of America; Copenhagen University Hospital at Herlev, DENMARK

## Abstract

For adoptive cell transfer (ACT) immunotherapy of tumor-reactive T cells, an effective therapeutic outcome depends upon cell dose, cell expansion *in vivo* through a minimally differentiated phenotype, long term persistence, and strong cytolytic effector function. An incomplete understanding of the biological coupling between T cell expansion, differentiation, and response to stimulation hinders the co-optimization of these factors. We report on a biophysical investigation of how the short-term kinetics of T cell functional activation, through molecular stimulation and cell-cell interactions, competes with phenotype differentiation. T cells receive molecular stimulation for a few minutes to a few hours in bulk culture. Following this priming period, the cells are then analyzed at the transcriptional level, or isolated as single cells, with continuing molecular stimulation, within microchambers for analysis via 11-plex secreted protein assays. We resolve a rapid feedback mechanism, promoted by T cell—T cell contact interactions, which strongly amplifies T cell functional performance while yielding only minimal phenotype differentiation. When tested in mouse models of ACT, optimally primed T cells lead to complete tumor eradication. A similar kinetic process is identified in CD8^+^ and CD4^+^ T cells collected from a patient with metastatic melanoma.

## Introduction

Adoptive cell therapy (ACT) of tumor-reactive T cells is being developed as a potentially curative treatment for patients with advanced cancer[1, 2]. In one approach, tumor antigen-specific T cells are expanded from tumor-infiltrating lymphocytes (TILs). In other cases, the T cells may be genetically modified to express cancer-specific receptors. The T cells are expanded ex vivo, and infused back into the patient. Various mouse model and clinical studies have been carried out to determine the optimal characteristics of this cellular infusion product [3–5]. Several factors appear important including cell dose, a strong cytolytic effector function, and the long-term persistence of the transferred cells [3, 4]. This latter characteristic has been linked to having a minimally differentiated phenotype, so as to maintain the capacity to produce a continual supply of cytolytic effector progeny. Simultaneous optimization of these factors is challenging, due to a not-well-understood biological coupling between T cell expansion, effector functions, terminal differentiation, and secondary response to different stimulations.

For mouse models of ACT, a 7–10 day period of *in vitro* stimulation and incubation of tumor-antigen specific T cells can lead to the increased production of cytotoxic granules, but can also yield terminally differentiated T cells[3, 4]. The net effect is a much reduced *in vivo* anti-tumor activity. The incubation period involves more than just a time period for the generation of a biological response to the stimulation. It also provides an environment conducive to T cell—T cell and T cell—antigen presenting cell (APC) interactions. These interactions can promote the formation of an immunological synapses[6, 7] and facilitate polarization of surface proteins[8] and directed cytokine secretion[9, 10]. T cells can also form clusters around APCs, which can promote synapse-mediated exchange of interleukin (IL)-2 between activated T cells[11]. Blocking adhesive interactions between stimulated T cells can diminish interferon-γ (IFN-γ) secretion[12]. Extended antigen exposure has also been implicated in promoting T cell phenotype differentiation, and down regulation of CD8 and TCR expression.[13]

The heterogeneous properties of bulk cell populations can mask the distinct molecule-cell and cell-cell interactions that influence T cell activation and subsequent anti-tumor effector properties. We carried out single cell biophysical kinetic experiments designed to separate the influence of these different interactions on T cell differentiation and gain of cytolytic effector function following *in vitro* stimulation. The kinetic period studied (0–24 hours post stimulation) is 10–100 fold shorter than that studied by most established protocols, and provides a high resolution view of how T cell biology evolves from baseline.

**Fig 1:**
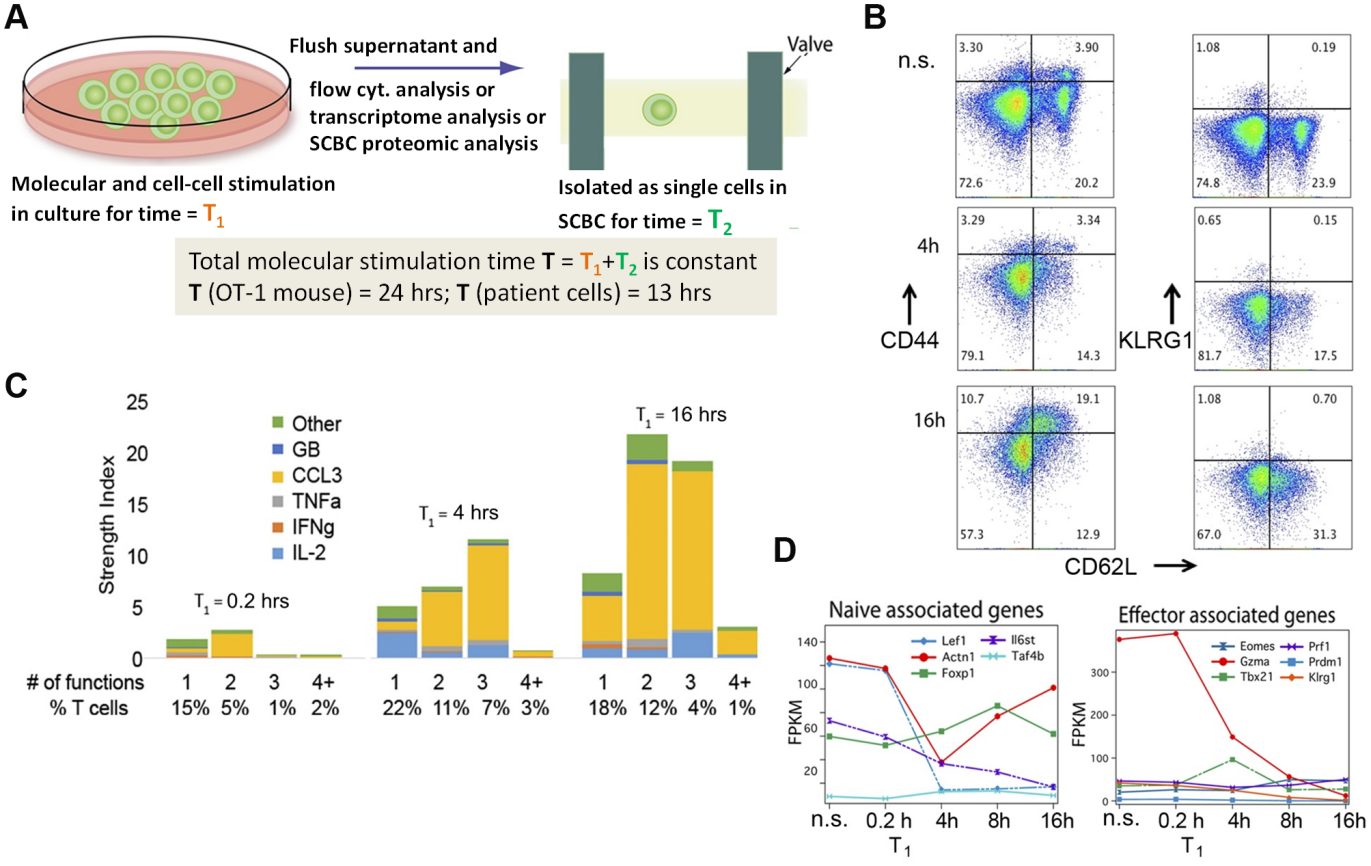
The short-time kinetics of OT-1 TCR-specific CD8+ T cell functional activation and phenotype differentiation. **A**. Experimental design. T cells are stimulated in bulk culture for a time period T_1_, during which those cells interact with each other. The supernatant is then flushed, and the cells are analyzed for phenotype using multi-color flow cytometry, transcriptome analysis, or they are resuspended and isolated, in the presence of molecular stimulant, within the microchambers of an SCBC, for a time T_2_. During T_2_, secreted proteins are captured by antibody arrays patterned within each microchamber. The total molecular stimulation time (T = T_1_ + T_2_) is constant. **B**. Protein secretion levels from single OT1 antigen-specific CD8+ T cells as T_1_ is increased from 10 minutes (0.2 hrs) to 16 hours. The y-axis is the pSI, calculated for cells secreting 1, 2, 3, or more proteins, out of 11 measured. **C**. Flow cytometry measures of the expression of CD62L vs. CD44 or KLRG1 as T_1_ increases from non-stimulated (n.s.) to 4 hrs to 16 hrs. **D**. The expression levels of naïve-associated and effector-associated genes as a function of increasing T_1_.

A protocol was designed to resolve the separate kinetics of T cell functional activation and T cell phenotypic differentiation following stimulation, and to quantitatively elucidate how T cell—T cell interactions influence those kinetics (Fig 1A). The protocol starts with molecular stimulation of the cells, and ends 24 hours later. The 24 hours are split into two time periods, T_1_ and T_2_. T_1_, which is varied from 10 minutes (0.2 hours) to 16 hours, is a priming period designed to promote T cell—T cell interactions, and to heighten their potential as anti-tumor effector cells. During T_1_, cells are incubated in bulk with continuing molecular stimulation. Transcriptome analysis, or cell phenotyping via multi-color flow cytometry, was carried out following T_1_. During T_2_, cell-cell interactions are stopped. The cells are washed, transferred to fresh medium, and then, with continuing molecular stimulation, isolated as single cells within the microwells of a single cell barcode chip (SCBC)[14, 15]. During T_2_, secreted proteins are captured by 11-plex antibody arrays patterned within each of ~1500 1 nanoliter volume microchambers within an SCBC (see Methods). Autocrine signaling is possible during T_2_, of course. By varying T_1_, the kinetics and extent to which homotypic T cell interactions influence the functional or phenotype evolution of stimulated T cells is resolved. During T_2_, the effect of the T_1_ priming period is functionally evaluated.

We used this 24 hour kinetic study to identify an optimized set of T_1_ priming protocols for preparing a T cell ACT infusion product, which was then tested in vivo using a mouse model of ACT. We then carried out a similar *in vitro* kinetic study of CD4+ and CD8+ T cells extracted from the peripheral blood of a melanoma cancer patient. A model for describing the short-term kinetics of T cell activation is provided.

## Results

### Strong functional enhancement of OT1 TCR-specific CD8^+^ T cells is promoted during the T_1_ priming period

Single cell proteomic and bulk transcriptomic kinetic studies were done on CD8+ tumor-antigen specific OT1 T cells isolated from the splenocytes of OT1 TCR transgenic mice. These cells were given a molecular stimulation of MHC class I tetramers loaded with the OVA peptide (the target of the OT1 TCR) and the anti-CD28 antibody. The influence of increasing T_1_ from 0.2 hours to 16 hours on the functional performance of OT-1 TCR-specific CD8^+^ T cells is shown in Fig 1b. For these plots, the T cell functionality is characterized using the polyfunctional strength index (pSI) metric, which emerged from an investigation of T cells infused into patients during an ACT therapy of TCR-engineered T cells. We found that those T cells that secreted multiple different proteins (i.e. were polyfunctional) also secreted, by far, the highest copy numbers of any given protein. This prompted the definition of the pSI[14], which is the fraction of cells secreting a certain number of different proteins multiplied by the measured intensity of those proteins. To permit direct comparisons of pSI values across different experimental conditions, the assayed protein levels were normalized by the time window (T_2_) allowed for protein secretion.

For T_1_ = 0.2 hours, only 23% of the T cells generate detectable secretion signal, and the average level of those secreted proteins is about 2-fold above background, excepting CCL3, which is almost 10-fold above background. After a 4 hour T_1_ period, around 43% of the single cells secrete at least one cytokine, and IL2 secretion signal is increased by ~10-fold. As T_1_ is increased to 16 hours, this trend is maintained, with increasing numbers of polyfunctional cells and copy numbers of secreted proteins (Fig 1B). These data strongly suggest that T cell-T cell interactions enhance the functionality of T cells well beyond what is achieved through molecular stimulation alone, and that this effect emerges within a few hours following molecular (antigen) stimulation.

### The T_1_ priming period promotes minimal differentiation of OT1 TCR-specific CD8^+^ T cells

The phenotype evolution of the OT-1 TCR specific CD8+ T cells across T_1_ was recorded via analysis of surface markers using flow cytometry (Fig 1C). Prior to stimulation, these cells contained a large percentage of naïve (CD62L+, CD44-) T cells. CD62L is an adhesion molecule normally expressed in naïve and younger type (e.g. central memory) T cells. Upon stimulation, the fraction of T cells expressing CD62L was largely unchanged, although the expression level of CD62L decreased (also S1 Fig), which is an expected outcome of antigen exposure [16]. CD44 is associated with activation and memory formation[17], and increased over the 16 hour T_1_ period. By contrast, the surface marker KLRG1, which is expressed in late stage effector T cells and implies replicative senescence and T cell exhaustion[16, 18], did not change over the 16 hour period. These data suggest that a conditioning period of up to 16 hours does not promote T cell differentiation to a late effector or exhausted phenotype.

The kinetics of gene expression for a few representative genes are shown in Fig 1D. A more in-depth transcriptome analysis is provided in S2–S6 Figs. For T_1_ = 10 min, the transcriptome of the stimulated cells resembles that of non-stimulated (control) cells (S3a Fig). As T_1_ increases from 0.2 hrs to 4 hrs, markers for T cell activation and cytokine genes are both upregulated (Panels B-F in S2 Fig), confirming the assay validity. The most strongly up-regulated genes with increasing T_1_ are transcription factors and biological processes important for T cell activation, differentiation, proliferation and memory maturation (S3 and S4 Figs)[19].

We further studied the kinetics of two subgroups of genes that have been suggested to be up-regulated (Panels A and B in S5 Fig) and down-regulated (Panels C and D in S5 Fig) in comparison of effector CD8^+^ T cells versus memory CD8^+^ T cells[20]. As T_1_ increases, we found that different genes within each subgroup move in opposite directions, suggesting a coexistence of effector development and memory maturation. The genes changing in accordance with effector development include enriched transcription factors and biological functions that are largely associated with the cell cycle, while the genes that changed in accordance with memory maturation are largely associated with T cell activation, regulation of memory formation, motility and membrane organization (S6 Fig)[21]. Certain transcripts, which have been shown to be indicators of T cell phenotype maturation[22], such as eomesodermin (controls cytolytic development of CD8^+^ T cells)[23, 24], T-bet[25], PR domain-containing 1 with ZNF domain[26], granzyme A and perforin, and KLRG1, either decreased or remained largely unchanged (Fig 1D). By contrast, certain transcripts associated with naïve T cells were partially decreased, or even slightly up-regulated (Fig 1D). Like the surface marker assays, these data demonstrate that the T_1_ conditioning period, although leading to highly functional T cells, does not promote significant T cell differentiation towards late effector phase or exhaustion.

### OT1 TCR-specific CD8+ T cells activated through the T_1_ priming period exhibit strong in vivo tumor killing performance

**Fig 2:**
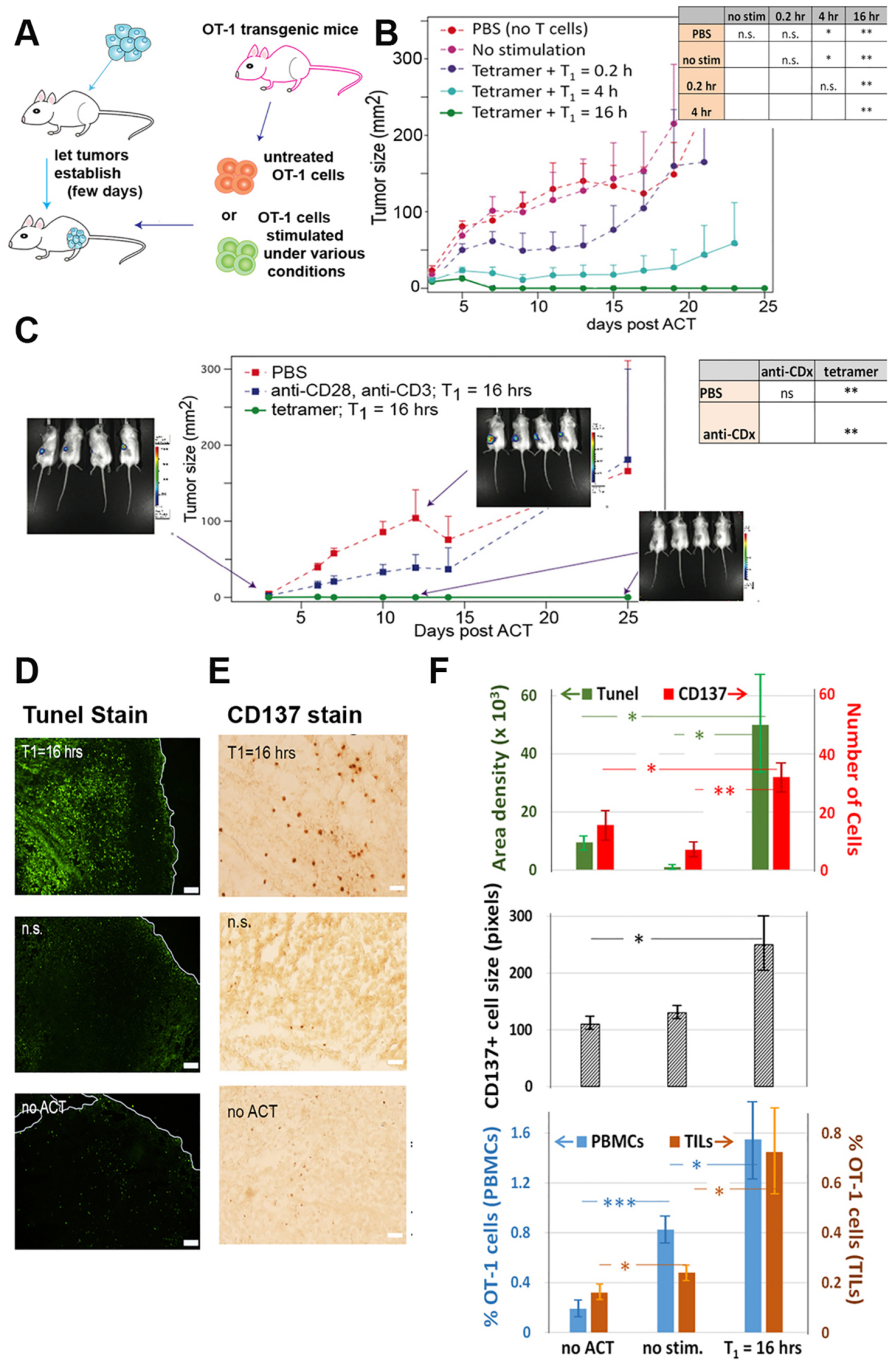
*In vivo* antitumor efficacy as a function of T_1_ and molecular stimulation in an ACT immunotherapy model. **A**. The mouse model ACT regimen that uses OT1 tumor antigen specific CD8^+^ T cells. **B**. Tumor growth curve (mean with error bars as s.e.m.) with the influence of the T_1_ priming period. Five conditions are shown: a buffer solution control (PBS), T cells with no stimulation, and T cells with molecular stimulation (OT1 tetramer + anti-CD28) for varying T_1_ periods. The figure represents one independent experiment with 5 mice per condition. The data are representative of two to three independent experiments (9–13 mice total per condition) with similar results. The inset chart provides statistical measures of the responses for the different conditions (n.s. = not significant, ** P < 0.005, * P < 0.05) **C**. Comparison between weak (anti-CD28 + anti-CD3) and strong (OT1 tetramer + anti-CD28), with a constant T_1_ (= 16 hours) prior to ACT. PBS is the negative control. Representative luciferase images of tumor at day 3 following ACT, at day 12 for the negative control and at day 12 following ACT for the strong stimulation condition. Similar images, for strong stimulation, and showing the absence of tumor, we collected at day 25. (Enlarged versions of these images are provided in S13 Fig). The inset chart provides statistical measures of the responses for the different treatment conditions, as assessed by a Kruskal-Wallis non-parametric comparison (n.s. = not significant, ** P < 0.005, * P < 0.05). This figure represents one independent experiment with 4 mice per condition, and is representative of two to three independent experiments (8–13 mice total per condition) with similar results. (**D-E**) Representative images of fluorescent Tunel tissue staining (**D**) and CD137 staining (**E**) assay 4 days after ACT. (top) ACT with OT1 T cells with a 16-hour T_1_ plus OT1 tetramer + anti-CD28 stimulation; (middle) with OT1 T cells with no stimulation; and (bottom) negative control (no ACT). The white line in (**D**) delineates the periphery of the tumor. Scale bar is 100 μm for Tunel staining and 50 μm for CD137 staining. **F**. Quantitation of staining and flow cytometry assays. (top) An intensity threshold is used to quantify Tunel staining of EG7 tumor cells (green bars), which is plotted with the average number (red bars) and size (middle graph) of CD137^+^ cells. Each histogram was based on 4 fields per section (*n* = 4–6 histological sections per animal; 4–5 animals per group). (bottom) Flow cytometry analysis of OT1 T cells *in vivo*. The percentage of CD8^+^ OT1 T cells among T cells in PBMC (blue) and in TILs (orange) are displayed. The x-axis labels apply to all plots. Values plotted are mean ± s.e.m (* *P* < 0.05, *** *P* < 0.001).

The in vitro kinetics data indicate that the priming regimen may be harnessed to exert a strong *in vivo* anti-tumor activity. We thus carried out ACT experiments in the EG.7 tumor model (Fig 2A–2C) engineered to provide antigen targets for the OT1 T cells. Following 2 days of tumor growth, CD8^+^ OT1 T cells that had experienced varying periods of T_1_ priming were adoptively transferred into the tumor bearing mice. Mice infused with cells that were stimulated with only a 10-minute T_1_ period exhibited tumor growth rates only slightly below the untreated controls. Increasing T_1_ from 10 minutes to 4 hours, and then to 16 hours led to increasing amounts of anti-tumor activity, with complete rejection of the tumors in all mice studied (n = 13 in three independent experiment rounds) for the cells with T_1_ = 16 hours (Fig 2B and 2C).

The 16 hours T_1_ priming exhibited improved antitumor activity relative to an alternative procedure designed for similarities to the established protocol [20, 27]. For this alternative procedure, the relevant antigen is added as a peptide, and then presented by antigen-presenting cells, rather than as an MHC tetramer. Specifically, cells are treated for 16 hours with peptide antigen (here OVA), plus IL2 stimulation of the splenocytes (S7 Fig). The splenocytes contain antigen-presenting cells. The S7 Fig data imply that a protocol that permits a limited period of T cell-T cell interactions following stimulation with MHC-presented antigen, can significantly optimize T cell activation for tumor killing.

We sought evidence of active tumor eradication via immunohistochemical (IHC) assays of tumor tissues and flow cytometry analysis of TILs (Fig 2D–2F). To ensure sufficient tumor materials for analysis, purified OT1 CD8^+^ T cells without stimulation, or with 16-hour T_1_ priming were adoptively transferred to mice a week after tumor injection when tumor diameters were >5 mm. For models treated with primed T cells, hematoxylin stains for gross cellular morphology demonstrated increased numbers of shrunken, apoptotic cells with pyknotic and fragmented nuclei, and condensed cytoplasms (S8 Fig). The level of apoptosis was compared across treatment conditions with a fluorescent Tunnel assay (Fig 2F (top panel, green bars)). Significant elevation of apoptotic cells was detected only in mice treated with primed T cells. Untreated tumors, or tumors treated with non-stimulated (n.s.) T cells, appeared similar. An IHC assay for the Ki67 cellular proliferation marker showed that tumors treated with the conditioned T cells exhibited reduced numbers of proliferating cells (S9 Fig). CD137 is a biomarker for activated CTLs[28],[29]. We observed elevation of both number (Fig 2F top panel, red bars) and size (Fig 2F middle panel) of CD137^+^ cells in mice treated with T_1_ = 16 hours primed T cells (S10 Fig). Untreated tumors, or tumors treated with n.s. T cells, appeared similar. We also observed a significant increase in the numbers of CD8^+^ OT1 T cells both in circulation (PBMCs) and TILs when we employed a 16-hour T_1_ conditioning regimen (Fig 2F and S11 Fig). These results imply that, following ACT, *in vitro* optimally primed T cells expand *in vivo*, migrate to the tumor site and directly kill tumor cells[30, 31].

To explore whether stimulation strength would also affect the tumor killing efficiency, we adoptively transferred OT1 T cells stimulated with just anti-CD3 and anti-CD28 antibodies. This weaker stimulation yields lower cytokine secretion during the T_1_ = 16 hrs priming (S12 Fig) and no significant anti-tumor activity (Fig 2C and S13 Fig). Collectively, these data suggest that effective priming depends on both the incubation time to promote T cell-T cell interactions, and on molecular stimulation strength. Such effective conditioning directly leads to enhanced tumor eradication.

### Human T cells respond similarly to similar conditioning regimens

**Fig 3:**
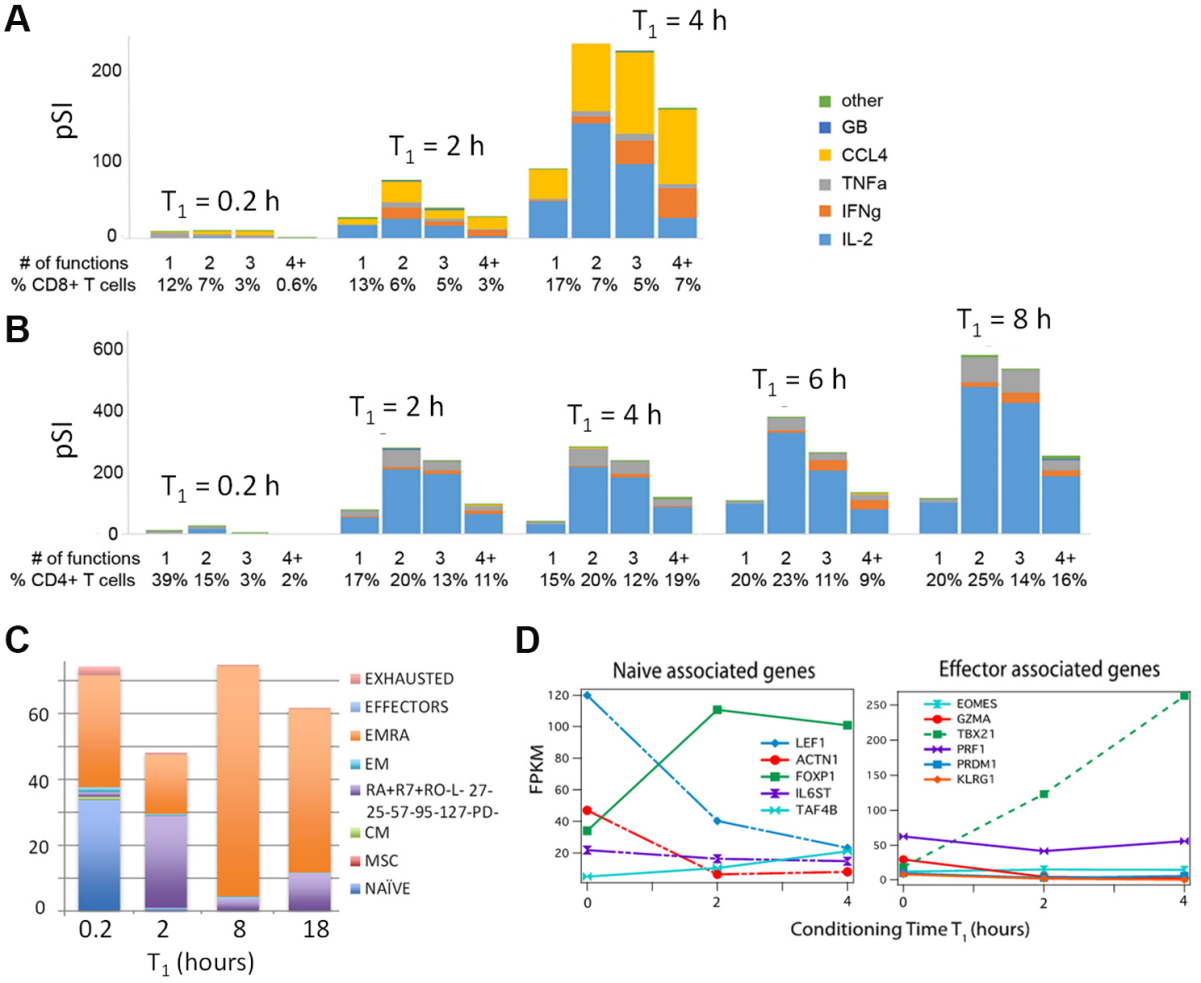
The effect of T_1_ priming on T cells extracted from the peripheral blood of a melanoma cancer patient. Strong stimulation (PMA + ionomycin + anti-CD23 + anti-CD28) over a variable T_1_ period increases the functionality and polyfunctionality of (**A**) CD8^+^ and (**B**) CD4+ T cells. The total time T = T_1_+T_2_ is held constant at 13 hours. **C**. The percentage of different phenotypes (described in detail in Materials and methods) as a function of T_1_ incubation period analyzed from multi-color flow cytometry of CD8^+^ T cells shows loss of the naïve phenotype, but no evidence of exhaustion or terminal differentiation. **D**. The expression level of naïve-associated and effector-associated genes as a function of T_1_ for CD8+ T cells.

We sought to explore the generality of the above results by investigating the kinetic response of human CD8^+^ and CD4^+^ T cells to a similar conditioning regimen (Fig 3). PBMCs were collected via leukapheresis from a patient with metastatic melanoma who had been selected for an ACT trial. Those cells represented a baseline for that patient, and were selected because they exhibited a phenotypic distribution (mostly naïve and effector memory RA (EMRA); Fig 3C) that was reasonably close to the OT1 TCR-specific CD8+ T cells analyzed above. A difference for the patient cell studies was that T_1_+T_2_ was held constant at the shorter period of 13 hours. This shorter time was selected because initial experiments indicated that the polyfunctional activation of the human T cells, under strong molecular stimulation, was faster than the mouse cells. Thus, shorter priming periods were selected so as to minimize T cell differentiation.

For the CD8+ T cells, T_1_ was varied from 0.2–4 hours, with a corresponding variation in T_2_ from 13–9 hours. These patient T cells were given a (standard) strong stimulation regimen of PMA, ionomycin and anti-CD23 and anti-CD28 antibodies. Similar to the OT1 T cell conditioning studies, we recorded a strong increase in T cell functional performance with increasing T_1_. The resultant cytokine profile closely resembles that of the OT1 T cells. IL2 and CCL4 (closely correlated with CCL3[14]) are the most dominant secretion products (Fig 3A).

Even more pronounced priming effects were observed for CD4^+^ T cells (Fig 3B). When T_1_ is extended stepwise from 0 to 8 hours, the production of secreted proteins increases more than 200 fold, and the fraction of polyfunctional T cells sharply increases. Interestingly, the relative percentages of the secreted proteins remain stable with increasing T_1_ (S14 Fig). We performed an accompanying kinetic analysis of the transcriptional profile. Similar to the case for the OT1 T cells, we found that, as T_1_ increases, the most strongly up-regulated genes are enriched transcription factors and biological processes critical for T cell activation, differentiation, proliferation and memory maturation (S15 and S16 Figs). A similar pattern of coexistence of effector development and memory maturation is also observed here for human T cells (S18 and S19 Figs). Moreover, we found that gene expression of CD8^+^ and CD4^+^ T cells are strongly correlated, with a correlation coefficient 0.96 for the non-stimulated state and 0.94 as T_1_ increases to 4 hours. A T_1_ priming period of a few hours did not yield effector or exhausted T cell phenotypes. This was determined via FC analysis of surface markers (Fig 3C shows results for CD8^+^ T cells; for CD4^+^ cells see Panel A S20 Fig) and in an analysis of transcripts associated with the expression of naive and effector genes (Fig 3D shows results for CD8^+^ T cells; for CD4^+^ cells see Panel B S20 Fig).

These data indicate that the patient T cells collected from peripheral blood respond to the priming regimen in a manner similar to the OT1 mouse model T cells. In other words, a strong stimulation of T cells over a few hour incubation period to promote T cell—T cell interactions, appears as a general approach for increasing the functional performance of T cells without significantly compromising their phenotype.

### The importance of T cell clustering during conditioning

We observed significant aggregation of T cells for both OT1 and human T cells during the T_1_ priming period, and we found that molecular stimulation of isolated T cells yielded little functional activation (Fig 1 and S3 Fig). Moreover, from the RNA-seq analysis, we found that biological processes associated with regulation of cell motility and adhesion are enriched as T_1_ increases (S6 and S16 Figs). Here we further quantify this process.

**Fig 4:**
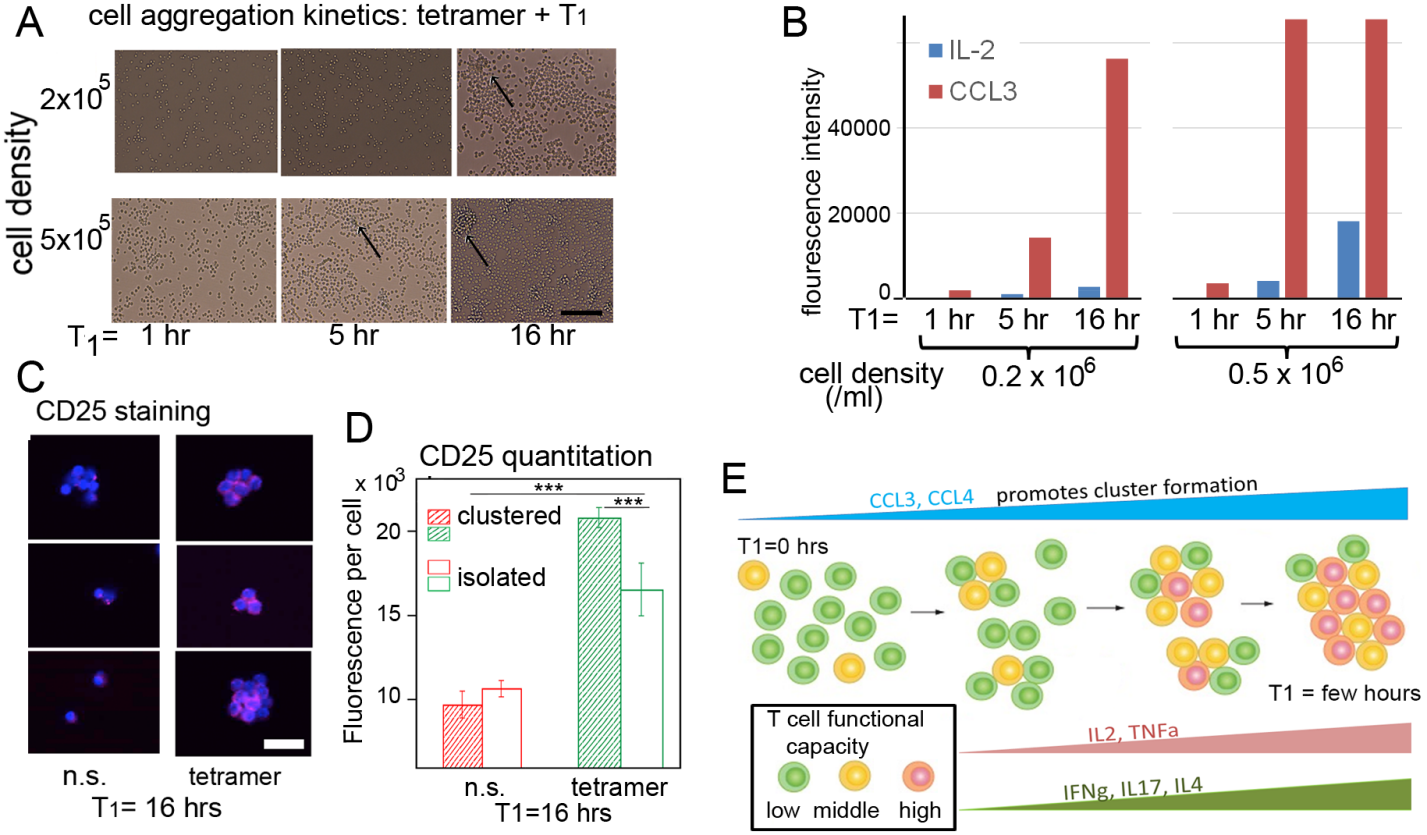
Correlations of T cell aggregation and T cell functionality increase during the T_1_ conditioning period, and a proposed associated mechanism. **A**. Micrographs showing the kinetics of the aggregation of OT-1 T cells following tetramer stimulation, over the course of T_1_, for cell densities of 2×10^5^ and 5×10^5^ cells/cm^3^. For the higher density culture, small, 2D cell aggregates are observed after T_1_ = 5 hours (arrow), while 3D aggregates are observed by T_1_ = 16 hours (arrow). At the lower cell density, 2D aggregates are observed by T_1_ = 16 hours (arrow). Scale bar = 200 μm. **B**. Dynamics of the production of the CCL3 and IL2 cytokines following tetramer stimulation of OT-1 T cells, as cell density is varied. In both cases, CCL3 production precedes IL2 production, and the production of both proteins ramps up more quickly at higher cell density. **C**. Fluorescent micrographs showing the staining of CD25 (the IL2 receptor, in red) for non-stimulated (n.s.) (left column) and tetramer (and CD28) stimulated OT-1 T cells (right column), after T_1_ = 16 hours. The cells were co-stained with the DAPI nuclear stain. Scale bar = 20 μm. **D**. Quantitation of the CD25 staining assays, measured in fluorescence intensity per cell (mean values ± s.e.m), using a threshold (*** *P* < 0.001, * *P* < 0.05). Statistics are based on 70–150 cells per condition. **E**. Drawing illustrating the dynamics of functional activation. The initial molecular stimulation promotes cell motility, via prompting CCL3 and CCL4 secretion. This, in turn, promotes increased contact interactions between T cells. Those interactions amplify the stimulation effects, leading to enhanced cell motility and additional contacts, in a fashion similar to a positive feedback loop. The feedback loop is established within the first one or two hours following molecular stimulation.

We altered the T cell aggregation kinetics by decreasing the cell concentration by 2- and 5-fold, relative to the 10^6^ /ml used for the above described priming experiments. For the lower densities, after one hour of stimulation, cells were largely isolated from each other (Fig 4A), and only CCL3 secretion was detected (Fig 4B; see S21 Fig for protein assay calibration data). After 5 hours of stimulation, a few contacting cells were seen in the low-density culture, and two-dimensional (2D) aggregates were seen in the higher density cell culture (Fig 4A). The higher density culture also exhibited a ~10 fold increase of IL2 and a >4-fold increase of CCL3 (the signal is saturated)) relative to the lower cell density culture. After 16 hours of stimulation, 2D aggregates are seen in both cultures, and 3D aggregates, along with 7-fold higher IL2 section, are seen in the higher density culture. These data suggest a positive coupling between cluster formation and amplification of cytokine secretion. This picture was supported by further investigations in which stronger molecular stimulation (with PMA and ionomycin) was used (S21 and S22 Figs).

IL2 amplification could be facilitated by IL2 signaling through IL2 receptors between active T cells [11, 32]. In fact, we observed a significant increase in the expression of the IL2 receptor alpha chain (CD25) as T_1_ increases in both OT1 and human T cells (S23 Fig). We also observed an increase in CD25 expression in OT1 T cells after 16-hr T_1_ priming with OT1 tetramer and CD28 (Fig 4C and 4D). Although CD25 expression is heterogeneous across individual cells, the mean fluorescence intensity integrated per cell is significantly enhanced within clusters compared to isolated cells after stimulation (Fig 4D). However, for cells with no stimulation, no significant difference was observed. This data further suggests that aggregation of stimulated T cells positively correlates with T cell functional activation, and that T cell interactions are important components of T cell activation.

## Discussion

In a previous adoptive immunotherapy clinical trial for melanoma using a MART-1 engineered T cell receptor (TCR)[33] or other TCR engineered ACT[34, 35], only transient results were observed. The addition of checkpoint inhibitors[36] may improve patient outcomes in such trials, but it is also worthwhile to investigate the quality of the transduced cells that are administered. There has been significant work in this area. For example, one suggestion has been to lower the high doses of IL-2 that are added to the cells in culture and to reduce the time (between 5 to 7 days) that those transduced cells spend in culture, as these conditions may create overdependence on the cytokines[5]. A second issue is cellular phenotype. The majority of the transduced T cells in the MART-1 engineered TCR trial were a mixture of effector memory and effector cells[14], while it has been described that naïve cells are superior for adoptive cell transfer therapy[37]. A third issue, which has been explored for both TCR-engineered[14] and CAR-T cell therapies[38] and recently shown to be important for CAR-T cell infusion products [39], is that of polyfunctionality. Here we studied a mouse model in which neither IL-2 nor long term culture of the adoptive transfer cells were necessary, and we started with a naïve phenotype.

Our approach was aimed at elucidating the separate roles that strong molecular stimulation and cell-cell interactions play in the short-term kinetics of T cell functional activation and phenotypic differentiation. A model of the T cell conditioning process is presented in Fig 4E. Previous studies have suggested that, upon stimulation, T cells secrete chemokine proteins such as CCL3 (and CCL4) that are associated with enhanced cell motility[40, 41]. In fact, these are the only proteins detected at short T_1_ conditioning times (Fig 4B, 1 hr). This suggests that an initial (short-time) consequence of molecular stimulation may be enhanced cell motility, which promotes aggregation (Fig 4A) and further activation. In fact, we find that, in the absence of T cell—T cell interactions following molecular stimulation, the functional activation of the T cells is arrested, even with continuing molecular stimulation. Other studies have shown that T cells aggregate around APCs to promote synapse-based exchange of cytokines[11, 12]. Consistent with this, we find that cytokine amplification and up-regulation of key cytokine receptors (e.g. IL2RA) is tightly coupled with the dynamics of cluster formation (Fig 4A–4D). Moreover, the enhanced cytokine secretion profile largely reflects an amplification of the secretion profile that is detected without training, but with a several-fold enhancement of the polyfunctional cells (Fig 3a and S14 Fig). We therefore hypothesize that the conditioning regimen exhibits an amplifying feedback: upon strong stimulation, increased motility, followed by aggregation, further increases stimulation. Based upon these kinetic studies, this process acts as a positive feedback loop with a doubling time constant of between one and a few hours, depending upon the T cell density and the molecular stimulation strength. We did not explore T_1_ times longer than 16 hrs (or 8 hrs for human cells). However, the T_1_ training periods explored here yielded a very large increase in the overall fraction of polyfunctional T cells. For example, the percentage of human CD8+ T cells that secrete 2 or more proteins increases from 20% to 55% with a T_1_ = 8 hours (Fig 3B). Longer T_1_ times are thus likely to yield diminishing returns on polyfunctionality, and can only promote T cell differentiation. As many clinical programs of CAR or TCR engineered T cell therapy include cryopreservation of the cellular product to perform lot release testing studies, followed by direct re-infusion to patients after conditioning chemotherapy, a straightforward application of the data presented herein would be to include an intermediate step of short term ex vivo culture with molecular stimulation, after thawing to foster T cell—T cell interactions resulting in a more functional set of cells re-infused to patients.

The fact that T cells exposed to very short (0.2 hr) T_1_ periods comprise a poor therapy (Fig 2B) implies that the infused T cells likely do not experience T cell contact interactions in vivo. This is consistent with the analysis of tumor tissues (Fig 2E) which show that the infiltrating CD137+ cells are not in contact.

T cells priming by dendritic cells (DCs) in lymph nodes *in vivo* has been described as three distinct phases[32, 42–44]. Initially, T cells enter the lymph nodes, searching for and transiently binding to DCs[45]. In the second phase, stable contacts between T cells and DCs are formed, accompanied by cytokine production and a dynamic clustering of T cells. In the third phase, T cells are released to migrate to the sites of infection, to proliferate, and to gain effector function. We hypothesize that our T_1_ conditioning mimics the first two phases, where both antigen and T cells are present in high density. The T_2_ period, in which the T cells are isolated, but exposed to molecular stimulant, represents a simplified model of phase three where T cells interact with the antigen in a relatively “isolated” fashion, perhaps imitating what happens at infection or tumor sites. The two-step T_1_ conditioning window followed by the T_2_ secretion period thus reveals a kinetic picture of how T cell-T cell interactions in the first two phases impact the fate and functional performance of T cells in the third phase. Thus, antigen exposure serves as a trigger for T cell activation, while T cell—T cell interactions largely amplify the functional consequences of that activation (Figs 1B, 3A and 3B). This functional activation is strongly regulated by stimulation strength, cell-cell interaction time and T cell phenotype (CD8^+^ or CD4^+^), but with a kinetics that is significantly faster than the kinetics of T cell terminal differentiation.

The amplitude of the cytokine secretion profile produced by isolated T cells during T_2_ is largely independent of T_2_, but depends upon the history of the T cells (the length of T_1_). This may point to the acquisition of a memory function, and is consistent with the phenotypic analysis. Moreover, the observed transcriptome dynamics during T_1_ revealed a coexistence of effector differentiation and memory development (S5, S6, S17 and S18 Figs), a property that is consistent with other studies [3]. This property could significantly benefit both rapid proliferation and long-term response *in vivo*. It has been proposed that T cell differentiation and functional enhancement are correlated with aging of phenotype [3]. A key finding here is that the functional performance and phenotype evolution proceed along different kinetic pathways, and thus may be temporally decoupled. Moreover, our *in vivo* results suggest that the short kinetic time window studied here could lead to enhanced capacity to expand *in vivo* (Fig 2F), to migrate to the tumor site (Fig 2F) and successfully eradicate the tumor (Fig 2B and 2C). All these attributes are important for the effectiveness of ACT. Proper utilization of this kinetic window, within a therapeutic context, therefore, may permit the optimization of T cell anti-tumor function without significantly changing the T cell phenotype. The results presented here are particularly relevant to ACT protocols involving T cell receptor engineered T cells, since the differentiation of those T cells is more subject to experimental control. The results reported here may not be as relevant to ACT protocols involves expanded tumor infiltrating lymphocytes.

## Materials and methods

### Clinical trial conduct

Patients were enrolled in the clinical trial after signing a written informed consent approved by the UCLA IRB (#08-02-020 and #10-001212) under an investigator new drug (IND) filed with the US Food and Drug Administration (IND# 13859). The study had the clinical trial registration number NCT00910650. Eligible patients had MART-1 positive metastatic melanoma by immunohistochemistry (IHC) and were HLA-A*0201 positive by intermediate resolution molecular HLA testing. This study was specifically approved by both the California Institute of Technology Committee for Protection of Human Subjects, and the University of California at Los Angeles Institutional Review Board (UCLA IRB).

### Mice and tumor cells

The murine models used were selected because of their previous use as models of ACT[46]. Female B6-albino mice (C57BL/6J mice that carry a mutation in the tyrosinase gene) and the OT1 TCR transgenic mice (C57BL/6-Tg(TcrαTcrβ) 1100Mjb/J) were purchased from the Jackson Laboratory. All mice were bred and housed in the Caltech animal facility according to institute regulations. All animal protocols were approved by the Caltech Institutional Animal Care and Use Committee. B6-albino mice were used at 7–10 weeks of age as recipient mice in the ACT experiments. EG.7 tumor cell line is derived from the tumor cell lines EL.4 (C57BL/6, H-2b, thymoma) with transfection of the chicken OVA cDNA. The cells were thawed from liquid nitrogen and cultured in C10 medium (RPMI with L-Gln (Corning 10-040-CV) supplemented with Pen-Strep, 10% (v/v) FBS, 10 mM HEPES, 50 μM beta-mercaptoethanol, 1x MEM NEAA and 1 mM sodium pyruvate) for a few days before injection. For each experimental condition, 4–5 mice were used for statistical rigor. All data are representative of two to three independent experiments (8–15 mice per condition). Statistics: tumor growth curves were assessed by two-way Analysis of Variance (ANOVA).

Mice were kept un-labeled until ACT of T cells treated with different condition is performed, allowing the mice to be randomly allocated to each conditioning group. Caliper measurements were assessed in blind by multiple investigators. The variation between groups was similar and expressed as mean with standard error (s.e.m).

### CD8^+^ T cell preparation and *in vitro* stimulation

The day before ACT, the spleen of the OT1 TCR transgenic mice (8 weeks to 6 months old) was harvested, and OT1 CD8^+^ T cells were purified from single-cell suspension of splenocytes using MACS column (Miltenyi Biotech) by depleting non-target cells (CD8^+^ T cells). Purified CD8^+^ T cells were then resuspended in cell culture medium (C10), at 1×10^6^ cells/ml in the 24-well plate. For tetramer stimulation: 10 μl tetramer (iTAg Tetramer/PE—H-2 Kb OVA (SIINFEKL), MBL) was added to 1×10^6^ T cells. Anti-CD28 antibodies were also added for a final concentration of 1 μg/ml. For anti-CD3 and anti-CD28 training: 1 μg/ml anti-CD3 antibodies were coated on the surface of 24-well plate for a couple of hours in the 37-degree incubator before T cells and anti-CD28 antibodies were added so the final concentration is 1 μg/ml. For both of the above stimulation conditions, we incubated the stimulated T cells at 37 degree CO_2_ incubator for a defined “training” time T_1_. In the peptide control, IL-2 (100 U/μl) and Ova peptide (1 μg/ml) were added directly to the single-cell suspension of splenocytes for 16 hours. After that, OT1 CD8^+^ T cells were purified using MACS column and then injected to the recipient mice. For human patient sample, T cells were stimulated with PMA (10 ng/ml), Ionomycin (1 μg/ml), anti-CD3 antibodies (2 μg/ml) and anti-CD28 antibodies (2 μg/ml).

### Adoptive cell transfer

E.G7 tumors were initiated by s.c. injection of 1×10^6^ cells into the left flank of the mice on day 0. At Day 2, treated or untreated CD8^+^ T cells were washed with PBS or medium once or twice to remove the supernatant and stimulants. Before injection, T cells were resuspended in PBS buffer at 10×10^6^ /ml and 100 μl (~ 1 million T cells total) were retro-orbitally (RO) injected to the recipient mice. Tumor size was measured every other day by using fine calipers (Manostat, Merenschwand, Switzerland) and is described as the product of the two largest perpendicular diameters (mm^2^). Mice were killed when the largest diameter of the tumor reached 20 mm.

Tumor growth was also monitored using the XENOGEN imaging device. The animals were anesthetized with 2–5% isoflurane, and injected with D-luciferin (15 mg/ml, 100 μl) and subsequently transferred to the imaging chamber, whereupon the isoflurane levels were reduced to 1–2.5%. The floor of the imager was heated to 37°C to avoid hypothermia. Breathing frequency was monitored and not allowed to drop below 1 s^−1^, adjusting the isoflurane levels accordingly at all times.

### Flow cytometry analysis of OT1 T cells

Flow cytometry was performed on a MACSQuant10 Analyzer (Miltenyi Biosciences). The cells were labeled with fluorescent antibodies specific for CD62L (Biolegend), CD44 (BD Biosciences) and KLRG1 (BD Biosciences).

### Integrated functional assays of single T cells

SCBCs were used to assay for a panel of 11 secreted proteins from individual T cells, using previously described experimental protocols[14, 15]. The volume for each microchamber is about 1 nanoliter (nl). For each SCBC chip, normally 300–400 chambers contain a single cell, and 200–300 chambers do not contain any cell. The other chambers contain more than one cell. Briefly, the surface of a DNA barcoded slide was blocked for 1 hour with 3% bovine serum albumin (BSA, Sigma) in PBS buffer (Irvine Scientific). This solution, and all subsequent solutions, was flowed at 1.5 psi for 1 hr with a total volume of 200 μl. A cocktail of antibody–DNA conjugates was then flowed onto the surface in order to hybridize them to the surface [15, 47]. T cells were stimulated for a set amount of time and loaded onto the SCBC chip at a cell density of 10^6^ cells/ml. The chip was video recorded using high-resolution bright field microscopy for cell counting purposes. T cells were incubated on chip for a varying amount of time at 37°C in a 5% CO_2_ cell incubator. Afterwards, the cells were washed out at 3 psi with 3% BSA for 10 min. The assay was completed by applying biotinylated antibodies and streptavidin-Cy5 and a final wash with 3% BSA to remove excess dye. Finally, the slide was washed with PBS and 50/50 PBS/deionized water before spin drying and scanning on a GenePix 4400A fluorescent scanner (Molecular Devices). Detailed calibration and validation has been supplied previously [15], where the measurement accuracy (coefficient of variation) of any given protein within a single cell assay is ~10%, and the assay sensitivity is several hundred molecules[15].

### Algorithm and statistics

Single cell data was gathered from two sources, 1) bright-field video for counting the numbers of cells within each microchamber of an SCBC; and 2) digitized images of the fluorescent sandwich-type immunoassay barcodes within those microchambers. The immunoassay fluorescence intensities were read using a GenePix 4400A scanner. These cell count and protein assay datasets were merged using a custom Matlab script. Each data set is comprised of assay results from around 300 0-cell microchambers and 400 1-cell microchambers. There are also around 350 2-cell and 200 3-cell, etc., microchambers. For each protein assay, a background signal level, for each barcode stripe, was calculated using the signal recorded from 0-cell microchambers. These background signals, when binned into 20 evenly spaced groups, could be fitted to a single Gaussian distribution (Kolmogorov-Smirnov test). For example, the background level of IFN-γ fit to a Normal distribution (mean = 106; std dev = 16.2) with a p-value of 0.58, which is well-above the threshold of 0.05. For 1-cell microchambers, the cutoff value for a cytokine-producing cell was defined as the mean of 0-cells plus twice the standard deviation. For some SCBCs, the background levels exhibited a gradient across the device that was corrected for in the analysis. For those microchambers containing 1 cell, each of the 11 protein assays were analyzed for evidence of secretion, as well as the level of that protein, recorded as a fluorescence intensity. These data were then used for the subsequent clustering and PCA analysis[48], as well as the analysis of the pSI of the cells. Statistics and visual presentation was automatically generated by the algorithm and was performed in Matlab software.

For the mouse model experiments in which tumor growth was monitored over time, for various treatment conditions, the growth curves were statistically compared using Kruskal-Wallis nonparametric tests.

### RNA-seq analysis

For each condition, 1×10^6^ T cells were washed twice with PBS solution. RNA was extracted and purified using the RNeasy Mini Kit (Qiagen). DNase treatment was then performed using the TURBO DNA-free kit (Ambion, #AM1907). Sequencing was performed by Illumina Hiseq2500 at Millard and Muriel Jacobs Genetics and Genomics Laboratory at Caltech. Sequencing reads were trimmed down to a uniform length (50bp) and aligned to the hg19 version of the human genome using TopHat version 2.0.8 and the GENCODE V16 transcription annotation as a reference for human T cells. For mouse, ENSEMBL 67 is used as a reference. Gene expression was quantified using Cufflinks version 2.0.2. All RNA-seq data files are publicly available at https://www.ncbi.nlm.nih.gov/Traces/study/?acc=SRP126680, accession: PRJNA422284.

### Methods and statistical analysis of immunohistochemistry

To ensure sufficient tumor materials for analysis, purified OT1 CD8^+^ T cells without stimulation or with 16-hour T_1_ priming were adoptively transferred to recipient mice 7 days after s.c. injection of 1×10^6^ EG.7 cells into the left flank. The tumors were collected, freshly frozen 4 days after ACT in tissue embedding medium O.C.T. compound (Scigen Inc., #4583) and stored at– 80°C. Tumors were transversally and sagitally sectioned in the middle (14 μm). The level of apoptosis was measured with a Tunnel assay using an *in situ* cell death detection kit (Roche, #11684795910) following manufacturer’s instructions. The following antibodies were used for IHC: anti-Ki67 (Abcam, USA, #ab16667), anti-CD137 (Abcam, USA, #ab197942), anti-CD25 (Thermo Fisher Scientific, USA, #MA5-12680). The quantification was based on 4 fields per section (n = 4–6 histological sections per animal; 4–5 animals per group). The fluorescence images were kept at the same exposure time for all conditions. The quantification of stained cell number, area and fluorescence intensity (with background subtraction) of IHC images were performed with ImageJ. Statistical analysis for IHC was performed using Prism 4.0b (Graphpad) and Matlab. Experiments with one variable factor were analyzed using one-way ANOVA, followed by a Bonferroni post hoc test. Experiments with two variable factors were subjected to two-way ANOVA, followed by a Bonferroni post hoc test and the corresponding *P* value was presented.

### *In vitro* T cell—T cell clustering analysis

CD8^+^ T cell preparation was conducted as described above. Purified CD8^+^ T cells were then resuspended in C10 medium, at various cell densities and stimulation conditions specified in the main text in the 96-well plate. For OT1 tetramer stimulation, 10 μl tetramer (iTAg Tetramer/PE—H-2 Kb OVA (SIINFEKL), MBL) and anti-CD28 antibodies (1 μg/ml)) were added to every 1×10^6^ T cells. The cell clustering was observed and the supernatant was collected for ensemble cytokine secretion analysis at different time points specified in the main text. For the analysis of CD25 expression, cells with a density of 1×10^6^ cells/ml were incubated with and without OT1 tetramer stimulation for 16 hours. The cells were then fixed with 2% paraformaldehyde and then the cell suspension was carefully transferred onto a glass slide. The CD25 expression was analyzed in single and clustered cells using anti-CD25 antibodies (Thermo Fisher Scientific, USA, #MA5-12680) after 16 h incubation. The cells were co-stained with DAPI. The quantification was done using an intensity threshold (ImageJ) and based on 70–150 cells per condition 4 fields per section.

### Human sample preparation and FACS analysis

#### PBMC sample procurement and processing

PBMC were isolated by Ficoll-Hypaque (Amersham Pharmacia, Piscataway, NJ) gradient centrifugation and cryopreserved in liquid nitrogen. Thawed PBMC were immediately diluted with RPMI complete media consisting of 10% human AB serum, and 1% penicillin, streptomycin and amphotericin (Omega Scientific), washed and subjected to enzymatic digestion with DNase (0.002%, Sigma) for 1 hour at 37°C. After removing the DNase, PBMC were incubated overnight at 37°C and 5% CO_2_ and stained for flow cytometry the next morning. For the SCBC analysis, PBMC were stained with the following markers for 30 minutes on ice: CD3 (HIT3a), CD4 (OKT4), CD8 (HIT8a), and 7-AAD for cell viability. Compensation Beads (BD Bioscience) were stained with the same colors to act as a reference. T cells were sorted on a FACS Aria II (BD Bioscience), gated on SSC-H, FSC-H, SSC-A, FSC-A, in addition to the surface markers mentioned above. Two populations were collected in the same vial: CD3^+^CD4^+^CD8^-^7-AAD^-^ and CD3^+^CD4^-^CD8^+^7-AAD^-^. The cells were washed 3x with media before stimulating them and loading onto the SCBC.

In the phenotype characterization assay (Fig 4C), PBMC were stained for CD3-BV650, CD4-BV510 and CD8-BV605, CD16-PC5, CD19-PC5 (Biolegend, CA, USA), and 7-aminoactinomycin 7AAD (Beckman Coulter, CA, USA) and sorted for live plus non-CD16+/non-CD19+/CD4+ and live plus non-CD16+/non-CD19+/CD8+ T cells on an FACS ARIAII and FACS ARIAIII.

#### Time course

After sorting, CD4^+^ and CD8^+^ T cells were centrifuged at 500g, 4 min, and resuspended at 1million per milliliter in X-Vivo-15 media, and incubated for 0, 2, 8 and 18h, in the following conditions: 1)-antiCD3 (OKT3) and anti CD28 (CD28.2) (eBioscience, Affymetrix, CA, USA), both at a final concentration of 2 μg/ml. 2)-Phorbol 12-myristate 13-acetate (PMA), and Ionomycin calcium salt from Streptomyces conglobatus (IONO) (both from Sigma Aldrich, MO, USA), both at a final concentration of 10ng/ml. 3) The combination of 1) and 2).

#### Flow cytometry surface staining and immunophenotyping

Sorted CD4^+^ and CD8^+^ T cells were centrifuged (500g for 4 minutes), resuspended in 100 μl of fetal bovine serum (Omega Scientific) to block F_**C**_ receptors, and stained with pre-conjugated antibodies for flow cytometry for 15 minutes at room temperature as described[49]. The reaction was stopped by adding 3 ml of phosphate buffered saline without calcium and magnesium (PBS) (Lonza, MD, USA). The pellet was centrifuged (500g for 4 minutes), resuspended in PBS, and the dead cell discriminator (7AAD) was added to the PBMC. All panel combinations of pre-conjugated antibodies were used at saturating conditions.

Panel 1 to 3 described T cell subset characterization, including activation and exhausted immune markers (see S1 Table). The fluorescence-minus-one (FMO) approach was used to gate positive and negative populations. We acquired between 50,000 of CD4^+^ and CD8^+^ T cells with an LSR II Flow Cytometer (BD Biosciences). Cells were described as exhausted if they co-expressed CD45RO+CD57+PD1+CD95+CD45RA–CCR7–CD62L–CD27–CD127–CD25+/–and Tim3+ cells. Effectors cells as: CD45RO+CD57+PD1–CD95+CD45RA–CCR7–CD62L–CD27+/–CD127–CD25+/–. EMRA cells: CD45RO–CD57+/–PD1+/–CD95+/–CD45RA+CCR7–CD62L+/–CD27+/–CD127+/–CD25+/–. Effector Memory cells: CD45RO+CD57–PD1–CD95+/–CD45RA–CCR7–CD62L–CD27+/–CD127+/–CD25+/–. Central memory cells: CD45RO+CD57+/–PD1+/–CD95+/–CD45RA–CCR7+CD62L+CD27+/–CD127+/–CD25+/–. Memory stem Cells: CD45RO+CD57–PD1–CD95+CD45RA+CCR7+CD62L+CD27+/–CD127+/–CD25+/–. Naïve cells: CD45RA+CCR7+CD62L+CD127+CD45RO–CD57–PD1–CD25+/–CD27+/–[22, 50–54].

#### Flow cytometry analysis

All flow data analyses were done with the FlowJo software (Tree Star Inc., Asland, OR, versions 9.6 and 9.7), and excel (Microsoft). The gating strategy is defined in S1–S3 Figs. [49]

## Supporting information

S1 Method Immunohistochemistry. (DOCX)

S2 Method Ensemble cytokine secretion detection. (DOCX)

S3 Method Tumor infiltrating lymphocytes (TIL) characterization. (DOCX)

S4 Method Flow cytometry analysis of mouse samples. (DOCX)

S5 Method RNA-seq analysis. (DOCX)

S1 Fig Phenotype dynamics of antigen specific OT1 CD8^+^ T cells. Histogram presentation of the expression level of surface markers CD62L, CD44 and KLRG1 as T_1_ increases from non-stimulated (n.s.) to T_1_ = 4 hours to T_1_ = 16 hours.
(DOCX)

S2 Fig Transcriptome dynamics of OT1 CD8^+^ T cells. **A**. A heatmap of the sequenced genes indicates a distinct and evolving gene expression profile as T_1_ increases. Color bar, mean (black) above (red) and below (blue) standard deviation. Representative cytokine genes including IL2 (B), CCL3 (C) and TNF (D) and surface marker for activation (CD69 (E) and CD44 (F)) are significantly up-regulated as T_1_ increases from 10 mins to 4 hours. The expression level of these genes between non-stimulated condition and 10 mins = T_1_ conditioning is largely unchanged, except for TNF.
(DOCX)

S3 Fig Enriched transcriptional network of OT1 CD8^+^ T cells from transcriptome analysis. A. Differential expressed genes relative to the 10 mins T_1_ conditioning are displayed with self-organizing map. In this map, each pixel represents a minicluster of genes. The organization of the map is based on all gene expression data sets (at all time points). Genes that exhibit very similar expression kinetics are grouped into the same or nearby miniclusters. Those genes with very different kinetics are mapped far apart from each other. The color of a pixel at a specific time point reflects the expression level of that minicluster at that time. B. Transcription factors that are enriched with the most strongly up-regulated genes as T_1_ is increases from non-stimulated to10 mins (upper), and from 10 mins to 16 hours (lower).
(DOCX)

S4 Fig Enriched biological processes of OT1 CD8^+^ T cells from transcriptome analysis as T_1_ is increased. The bar graphs show differences between 16 hrs and 10 min, and 16 hrs and 4 hrs.
(DOCX)

S5 Fig Gene dynamics of OT1 CD8^+^ T cells that are highly correlated with effector-vs-memory regulation. The dynamic change of genes up-regulated in comparison of effector CD8 T cells versus memory CD8 T cells as a function of T_1_ represented by heatmap (A) and GATE self-organizing map (B). The dynamics genes down-regulated in comparison to effector CD8 T cells versus memory CD8 T cells as a function of T_1_, represented by a heatmap (C) and a GATE self-organizing map (D).
(DOCX)

S6 Fig Enriched transcription factors (A) and biological processes (B) by genes that are regulated in the same way in comparison of effector CD8 T cells versus memory CD8 T cells as T_1_ increases. Enriched transcription factors (C) and Biological processes (D) by genes that are regulated in the opposite way in comparison of effector CD8 T cells versus memory CD8 T cells as T_1_ increases for OT1 T cells.
(DOCX)

S7 Fig *In vivo* antitumor efficacy with peptide control vs. selected conditions in Fig 2. For the peptide control, OVA peptide and IL2 were added directly to the splenocytes (details in Methods section), along with antigen-presenting cells. In the tetramer stimulation, tetramer and anti-CD28 were used as the molecular stimulation. Values plotted are mean ± s.e.m, with a statistical comparison between experimental conditions provided in the inset table (* *P* < 0.05, ** *P* < 0.005).
(DOCX)

S8 Fig Gross cell morphology of EG.7 tumor 4 days after ACT under various conditions. Hematoxylin staining demonstrates increased number of apoptotic cells that are shrunken with pyknotic and fragmented nuclei and condense cytoplasm after adoptive transfer of CD8^+^ T cells under 16-hour T_1_ conditioning with Ova tetramer and anti-CD28 stimulation (A) compared to non-stimulated CD8^+^ T cells (B) and without adoptive T cell transfer (C). Representative hematoxylin-stained sections are displayed. Bar, 20 μm.
(DOCX)

S9 Fig The level of proliferation in EG.7 tumor after 4 days after ACT under various conditions. Ki67 staining demonstrates decreased numbers of proliferating cells after adoptive transfer of CD8+ T cells under 16-hour T_1_ conditioning with Ova tetramer and anti-CD28 stimulation (A) compared to non-stimulated CD8+ T cells (B) and without adoptive T cell transfer (C). Representative examples are shown. Bar, 50 μm. (D) The proliferation index (mean ± s.e.m) was quantified using an intensity threshold. Analysis was done by one-way ANOVA followed by Bonferroni’s multiple comparison test (*:p < 0.05). The quantification was based on 4 fields per section (*n* = 4–8 histological sections per animal; 3–4 animals per group).
(DOCX)

S10 Fig Quantification of CD137 staining. The total area (means ± s.e.m) was quantified using an intensity threshold. Analysis was done by one-way ANOVA followed by Bonferroni’s multiple comparison test (**: *P* < 0.01). The quantification was based on 4 fields per section (*n* = 4–6 histological sections per animal; 4–5 animals per group).
(DOCX)

S11 Fig Population of OT1+ cells that infiltrate into the tumor site. Flow cytometric determination of OT1+ T cells in mouse tumor biopsy 4 days after ACT of OT1 T cells with 16-hour T_1_ conditioning with molecular stimulation (OT1 tetramer + anti-CD28), or no stimulation.
(DOCX)

S12 Fig Ensemble cytokine secretion during T_1_ = 16 h for different molecular stimulation (anti-CD3 + anti-CD28, left) and (OT1 tetramer + anti-CD28). (DOCX)

S13 Fig Enlarged luciferase images displayed in Fig 2C. (DOCX)

S14 Fig Relative number of cells that secrete a particular cytokine for human CD4^+^ T cells. The secretion profile is largely similar for various T_1_ conditioning time. The absolute number of cells that secrete and the average secretion intensity, however, are significantly increased as T_1_ increases (shown in the main text).
(DOCX)

S15 Fig Enriched transcriptional network of human CD8^+^ T cells from transcriptome analysis. A. Differential expressed genes relative to the 0-hour (NS) T_1_ conditioning are displayed with self-organizing map. B. Transcription factors that are enriched with the most dramatically up-regulated genes as T_1_ is increased from 0 hour (non-stimulated) to 2 hours.
(DOCX)

S16 Fig Enriched biological processes of human CD8^+^ T cells from transcriptome analysis as T_1_ is increased from 0 hour (NS) to 2 hours (A) and from 2 hours to 4 hours (B). (DOCX)

S17 Fig Gene dynamics of human CD8^+^ T cells that are highly correlated with effector-vs-memory regulation. The dynamic change of genes up-regulated in comparison of effector CD8 T cells versus memory CD8 T cells as a function of T_1_ represented by heatmap (A) and GATE self-organizing map (B). The dynamic change of genes down-regulated in comparison of effector CD8 T cells versus memory CD8 T cells as a function of T_1_ represented by heatmap (C) and GATE self-organizing map (D).
(DOCX)

S18 Fig Enriched transcription factors (A) and biological processes (B) by genes that are regulated in the same way in comparison of effector CD8 T cells versus memory CD8^+^ T cells as T_1_ increases. (DOCX)

S19 Fig Correlation of human (A) CD4^+^ and (B) CD8^+^ T cells from the same patient as T_1_ increases from 0 hour (NS) to 4 hours. (DOCX)

S20 Fig Phenotypic evolution and transcriptome dynamics for human CD4^+^ T cells after various T_1_ conditioning time. A. Phenotype analysis from multi-color flow cytometry of CD4^+^ T cells shows loss of the naïve phenotype, but no evidence of terminal differentiation. B. The expression level of naïve-associated (upper) and effector-associated (lower) genes as a function of T_1_ for CD4^+^ T cells.
(DOCX)

S21 Fig Cytokine secretion dynamics and calibration curve. A. Characterization of secreted cytokines (CCL3 (upper) and IL2 (lower)) for OT1 T cells with molecular stimulation (OT1 tetramer + anti-CD28 + PMA + Ionomycin) under different cell densities (higher: 5 × 10^5^/ml, lower: 2 × 10^5^/ml) as T_1_ increases. B. Calibration curve of fluorescence signal vs. protein concentration.
(DOCX)

S22 Fig Cell clustering dynamics with strong molecular stimulation (OT1 tetramer + anti-CD28 + PMA + ionomycin). Representative images of the clustering area under different cell densities (upper: 2 × 10^5^/ml, lower: 5 × 10^5^/ml). Scale bar = 200 μm.
(DOCX)

S23 Fig The RNA expression level of IL2 receptor (IL2RA) as a function of T_1_ conditioning time. A consistent elevation was observed for OT1 CD8^+^ T cells, human CD4^+^ and CD8^+^ T cells.
(DOCX)

S1 Table List of antibody panel used for flow cytometry immuno-phenotyping analysis for human patient sample. (DOCX)

S2 Table List of antibody panel for human T cells in single cell barcode chip (SCBC). (DOCX)

S3 Table List of antibody panel for mouse T cells in SCBC. (DOCX)
