# Supplementary material for: A kinetic investigation of interacting, stimulated T cells identifies conditions for rapid functional enhancement, minimal phenotype differentiation, and improved adoptive cell transfer tumor eradication
Source: PLoS One. 2018 Jan 23;13(1):e0191634. doi: 10.1371/journal.pone.0191634 (PMC5779691; doi:10.1371/journal.pone.0191634)
Supplement: S1 Method — (DOCX) [file pone.0191634.s001.docx]

**S1 Method. Immunohistochemistry**

To ensure sufficient tumor materials for analysis, purified OT1 CD8^+^ T cells without stimulation or with 16-hour T_1_ conditioning were adoptively transferred to recipient mice 7 days after s.c. injection of 1×10^6^ EG.7 cells into the left flank. The tumors were collected, freshly frozen 4 days after ACT in tissue embedding medium O.C.T. compound (Scigen Inc., #4583) and stored at - 80 °C. Tumors were transversally and sagitally sectioned in the middle (14 μm). Gross cell morphology was assessed on hematoxylin (Mayer’s)-stained sections. The level of apoptosis was measured with a Tunnel assay using an *in situ* cell death detection kit (Roche, #11684795910) following manufacturer’s instructions. Immunohistochemistry for a cellular marker for proliferation Ki67 was performed using anti-Ki67 (Abcam, USA, #ab16667). The fluorescence (Tunnel assay) and bright field (Ki67 and cell morphology) images were kept at the same exposure time for all conditions. The proliferation index for Ki-67 staining was done using ImageJS Ki67 module (<https://chrome.google.com/webstore/search/imagejs>) and based on 4 fields per section (n = 4-8 histological sections per animal; 3-4 animals per group).
