# Supplementary material for: A kinetic investigation of interacting, stimulated T cells identifies conditions for rapid functional enhancement, minimal phenotype differentiation, and improved adoptive cell transfer tumor eradication
Source: PLoS One. 2018 Jan 23;13(1):e0191634. doi: 10.1371/journal.pone.0191634 (PMC5779691; doi:10.1371/journal.pone.0191634)
Supplement: S2 Method — (DOCX) [file pone.0191634.s002.docx]

**S2 Method. Ensemble cytokine secretion detection**

After T_1_ conditioning, the supernatant with secreted protein was collected for ensemble proteomic analysis based on a DNA-encoded Antibody Library (DEAL) assay (*42*). Briefly, A DNA barcoded slide is sectioned into wells using molded elastomers. The surface of the slide was blocked for 1 hour with 3% bovine serum albumin (BSA, Sigma) in PBS buffer (Irvine Scientific). A cocktail of antibody-DNA conjugates was added and incubated for an hour in order to be hybridized them to the surface (*15, 42*). After washing the wells 3 times with 3% BSA, the supernatant containing the secreted proteins was added to individual wells and incubated for an hour. Afterwards, wells were washed again 3 times with 3% BSA. The assay was completed by applying biotinylated antibodies and streptavidin-Cy5 and a final wash with 3% BSA to remove excess dye. Finally, the slide was washed with PBS before spin drying and scanning on a GenePix 4400A fluorescent scanner (Molecular Devices).
