# Supplementary material for: A kinetic investigation of interacting, stimulated T cells identifies conditions for rapid functional enhancement, minimal phenotype differentiation, and improved adoptive cell transfer tumor eradication
Source: PLoS One. 2018 Jan 23;13(1):e0191634. doi: 10.1371/journal.pone.0191634 (PMC5779691; doi:10.1371/journal.pone.0191634)
Supplement: S3 Method — (DOCX) [file pone.0191634.s003.docx]

**S3 Method. Tumor infiltrating lymphocytes (TIL) characterization**

To ensure sufficient tumor materials for analysis, purified OT1 CD8^+^ T cells without stimulation or with 16-hour T_1_ conditioning were adoptively transferred to recipient mice 7 days after s.c. injection of 1×10^6^ EG.7 cells into the left flank. 4 days after ACT, the tumors were collected and cut into small pieces and incubated in 50 U/ ml DnaseI and 1.4 mg/ml collagenase in C10 medium (specified in the main text) for 30 minutes at 37 ºC. Cells in suspension were then passed through a 70 - µm Nylon cell strainer (BD Falcon). Lymphocytes were enriched through Percoll (GE Heatlthcare) gradient centrifugation following their protocol and then used for flow cytometry analysis.
