# Supplementary material for: A kinetic investigation of interacting, stimulated T cells identifies conditions for rapid functional enhancement, minimal phenotype differentiation, and improved adoptive cell transfer tumor eradication
Source: PLoS One. 2018 Jan 23;13(1):e0191634. doi: 10.1371/journal.pone.0191634 (PMC5779691; doi:10.1371/journal.pone.0191634)
Supplement: S4 Method — (DOCX) [file pone.0191634.s004.docx]

**S4 Method. Flow cytometry analysis of mouse samples**

Flow cytometry was performed on a MACSQuant10 Analyzer (Miltenyi Biosciences). The cells were labeled with fluorescent antibodies specific for CD45 (Biolegend), CD3 (eBioscience), OT1 tetramer (MBL) and CD8 (Biolegend).
