# Supplementary material for: A kinetic investigation of interacting, stimulated T cells identifies conditions for rapid functional enhancement, minimal phenotype differentiation, and improved adoptive cell transfer tumor eradication
Source: PLoS One. 2018 Jan 23;13(1):e0191634. doi: 10.1371/journal.pone.0191634 (PMC5779691; doi:10.1371/journal.pone.0191634)
Supplement: S5 Method — (DOCX) [file pone.0191634.s005.docx]

**S5 Method. RNA-seq analysis**

The RNA-seq analysis and visualization are represented by heat map (using Matlab software) or self-organized maps (using the Gene Expression Dynamics Inspector software (GEDI, <http://www.childrenshospital.org/research/ingber/GEDI/gedihome.htm>)(*1*) and the GATE software (<http://amp.pharm.mssm.edu/maayan-lab/gate.htm>))(*2*). The differentially expressed genes under different conditions are then used for enrichment analysis using the Transcription Factor PPIs and GO Biological Process gene set libraries on the Enrichr website (http://amp.pharm.mssm.edu/Enrichr/). Gene lists that are up-regulated or down-regulated in comparison of effector CD8 T cells versus memory CD8 T cells are obtained from GSEA (http://www.broadinstitute.org/gsea/msigdb/cards/GOLDRATH_EFF_VS_MEMORY_CD8_TCELL_UP.html).
