# Supplementary material for: A kinetic investigation of interacting, stimulated T cells identifies conditions for rapid functional enhancement, minimal phenotype differentiation, and improved adoptive cell transfer tumor eradication
Source: PLoS One. 2018 Jan 23;13(1):e0191634. doi: 10.1371/journal.pone.0191634 (PMC5779691; doi:10.1371/journal.pone.0191634)
Supplement: S1 Fig — Histogram presentation of the expression level of surface markers CD62L, CD44 and KLRG1 as T1 increases from non-stimulated (n.s.) to T1 = 4 hours to T1 = 16 hours. (DOCX) [file pone.0191634.s006.docx]

**S1 Fig. Phenotype dynamics of antigen specific OT1 CD8^+^ T cells.** Histogram presentation of the expression level of surface markers CD62L, CD44 and KLRG1 as T_1_ increases from non-stimulated (n.s.) to T_1_ = 4 hours to T_1_ = 16 hours.
