# Supplementary material for: A kinetic investigation of interacting, stimulated T cells identifies conditions for rapid functional enhancement, minimal phenotype differentiation, and improved adoptive cell transfer tumor eradication
Source: PLoS One. 2018 Jan 23;13(1):e0191634. doi: 10.1371/journal.pone.0191634 (PMC5779691; doi:10.1371/journal.pone.0191634)
Supplement: S2 Fig — A. A heatmap of the sequenced genes indicates a distinct and evolving gene expression profile as T1 increases. Color bar, mean (black) above (red) and below (blue) standard deviation. Representative cytokine genes including IL2 (B), CCL3 (C) and TNF (D) and surface marker for activation (CD69 (E) and CD44 (F)) are significantly up-regulated as T1 increases from 10 mins to 4 hours. The expression level of these genes between non-stimulated condition and 10 mins = T1 conditioning is largely unchanged, except for TNF. (DOCX) [file pone.0191634.s007.docx]

**S2 Fig. Transcriptome dynamics of OT1 CD8^+^ T cells. A.** A heatmap of the sequenced genes indicates a distinct and evolving gene expression profile as T_1_ increases. Color bar, mean (black) above (red) and below (blue) standard deviation. Representative cytokine genes including IL2 (B), CCL3 (C) and TNF (D) and surface marker for activation (CD69 (E) and CD44 (F)) are significantly up-regulated as T_1_ increases from 10 mins to 4 hours. The expression level of these genes between non-stimulated condition and 10 mins = T_1_ conditioning is largely unchanged, except for TNF.
