# Supplementary material for: A kinetic investigation of interacting, stimulated T cells identifies conditions for rapid functional enhancement, minimal phenotype differentiation, and improved adoptive cell transfer tumor eradication
Source: PLoS One. 2018 Jan 23;13(1):e0191634. doi: 10.1371/journal.pone.0191634 (PMC5779691; doi:10.1371/journal.pone.0191634)
Supplement: S3 Fig — A. Differential expressed genes relative to the 10 mins T1 conditioning are displayed with self-organizing map. In this map, each pixel represents a minicluster of genes. The organization of the map is based on all gene expression data sets (at all time points). Genes that exhibit very similar expression kinetics are grouped into the same or nearby miniclusters. Those genes with very different kinetics are mapped far apart from each other. The color of a pixel at a specific time point reflects the expression level of that minicluster at that time. B. Transcription factors that are enriched with the most strongly up-regulated genes as T1 is increases from non-stimulated to10 mins (upper), and from 10 mins to 16 hours (lower). (DOCX) [file pone.0191634.s008.docx]

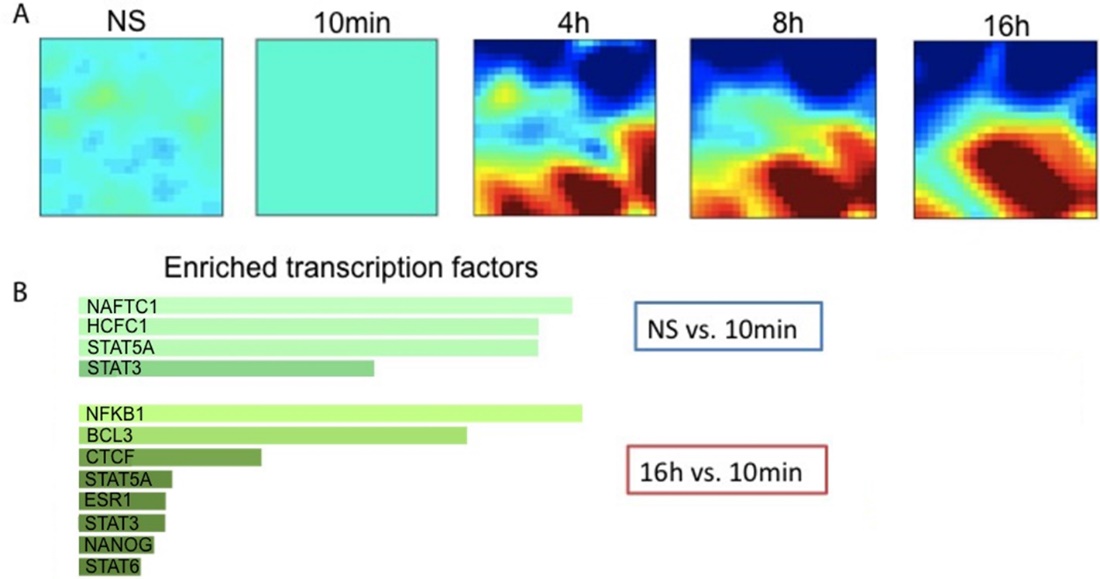


**S3 Fig. Enriched transcriptional network of OT1 CD8^+^ T cells from transcriptome analysis.** A. Differential expressed genes relative to the 10 mins T_1_ conditioning are displayed with self-organizing map. In this map, each pixel represents a minicluster of genes. The organization of the map is based on all gene expression data sets (at all time points). Genes that exhibit very similar expression kinetics are grouped into the same or nearby miniclusters. Those genes with very different kinetics are mapped far apart from each other. The color of a pixel at a specific time point reflects the expression level of that minicluster at that time. B. Transcription factors that are enriched with the most strongly up-regulated genes as T_1_ is increases from non-stimulated to10 mins (upper), and from 10 mins to 16 hours (lower).
