# Supplementary material for: A kinetic investigation of interacting, stimulated T cells identifies conditions for rapid functional enhancement, minimal phenotype differentiation, and improved adoptive cell transfer tumor eradication
Source: PLoS One. 2018 Jan 23;13(1):e0191634. doi: 10.1371/journal.pone.0191634 (PMC5779691; doi:10.1371/journal.pone.0191634)
Supplement: S4 Fig — The bar graphs show differences between 16 hrs and 10 min, and 16 hrs and 4 hrs. (DOCX) [file pone.0191634.s009.docx]

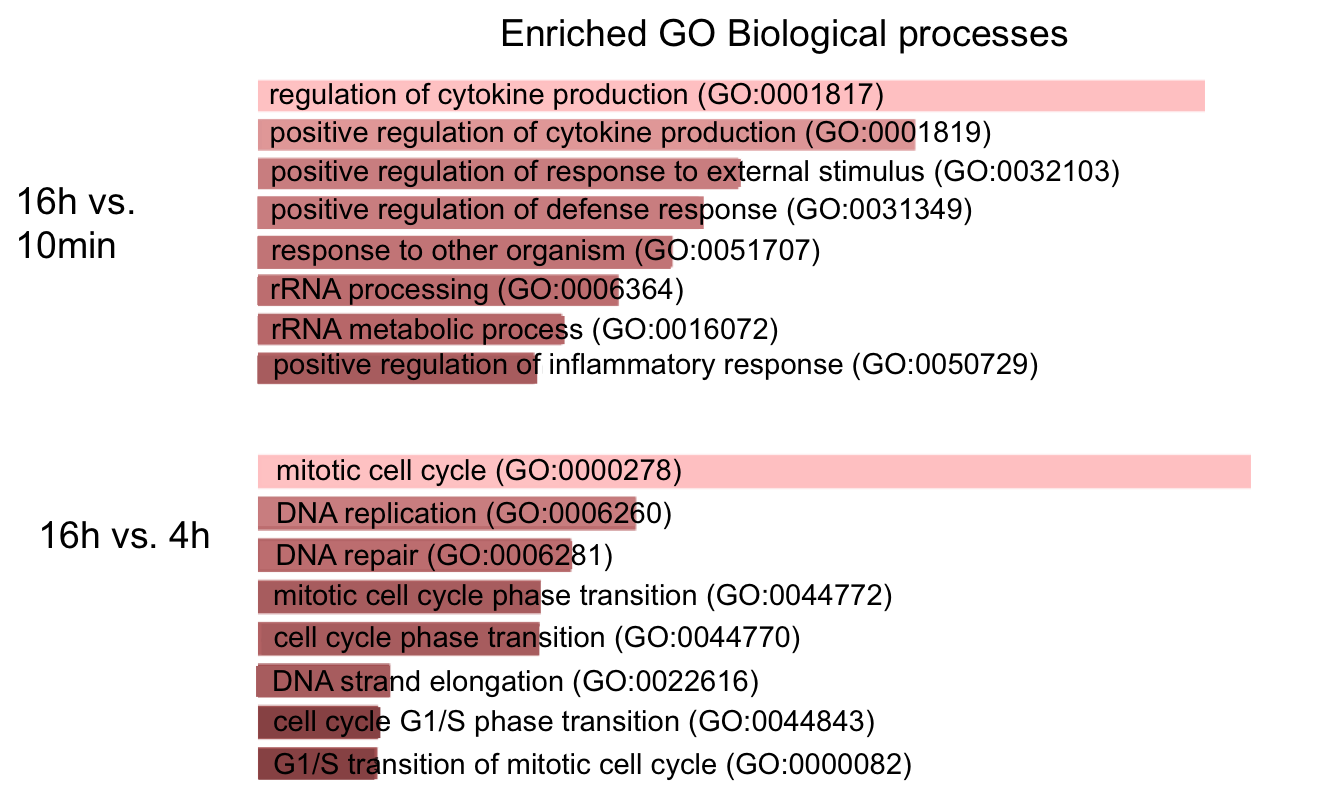


**S4 Fig.** Enriched biological processes of OT1 CD8^+^ T cells from transcriptome analysis as T_1_ is increased. The bar graphs show differences between 16 hrs and 10 min, and 16 hrs and 4 hrs.
