# Supplementary material for: A kinetic investigation of interacting, stimulated T cells identifies conditions for rapid functional enhancement, minimal phenotype differentiation, and improved adoptive cell transfer tumor eradication
Source: PLoS One. 2018 Jan 23;13(1):e0191634. doi: 10.1371/journal.pone.0191634 (PMC5779691; doi:10.1371/journal.pone.0191634)
Supplement: S5 Fig — The dynamic change of genes up-regulated in comparison of effector CD8 T cells versus memory CD8 T cells as a function of T1 represented by heatmap (A) and GATE self-organizing map (B). The dynamics genes down-regulated in comparison to effector CD8 T cells versus memory CD8 T cells as a function of T1, represented by a heatmap (C) and a GATE self-organizing map (D). (DOCX) [file pone.0191634.s010.docx]

**
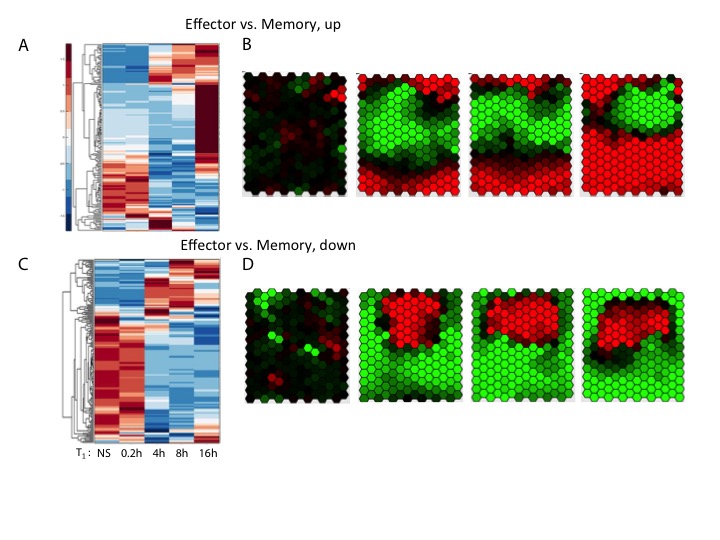
**

**S5 Fig. Gene dynamics of OT1 CD8^+^ T cells that are highly correlated with effector-vs-memory regulation.** The dynamic change of genes up-regulated in comparison of effector CD8 T cells versus memory CD8 T cells as a function of T_1_ represented by heatmap (A) and GATE self-organizing map (B). The dynamics genes down-regulated in comparison to effector CD8 T cells versus memory CD8 T cells as a function of T_1_, represented by a heatmap (C) and a GATE self-organizing map (D).
