# Supplementary material for: A kinetic investigation of interacting, stimulated T cells identifies conditions for rapid functional enhancement, minimal phenotype differentiation, and improved adoptive cell transfer tumor eradication
Source: PLoS One. 2018 Jan 23;13(1):e0191634. doi: 10.1371/journal.pone.0191634 (PMC5779691; doi:10.1371/journal.pone.0191634)
Supplement: S6 Fig — Enriched transcription factors (C) and Biological processes (D) by genes that are regulated in the opposite way in comparison of effector CD8 T cells versus memory CD8 T cells as T1 increases for OT1 T cells. (DOCX) [file pone.0191634.s011.docx]

**
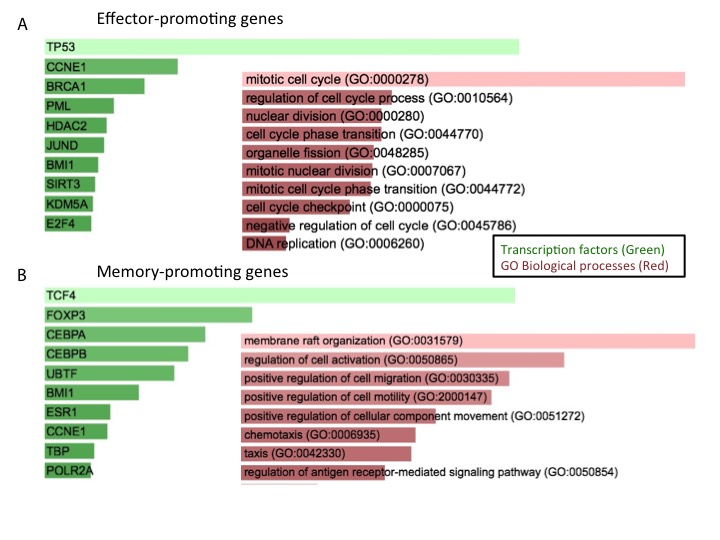
**

**S6 Fig. Enriched transcription factors (A) and Biological processes (B) by genes that are regulated in the same way in comparison of effector CD8 T cells versus memory CD8 T cells as T_1_ increases.** Enriched transcription factors (C) and Biological processes (D) by genes that are regulated in the opposite way in comparison of effector CD8 T cells versus memory CD8 T cells as T_1_ increases for OT1 T cells.
