# Supplementary material for: A kinetic investigation of interacting, stimulated T cells identifies conditions for rapid functional enhancement, minimal phenotype differentiation, and improved adoptive cell transfer tumor eradication
Source: PLoS One. 2018 Jan 23;13(1):e0191634. doi: 10.1371/journal.pone.0191634 (PMC5779691; doi:10.1371/journal.pone.0191634)
Supplement: S7 Fig — For the peptide control, OVA peptide and IL2 were added directly to the splenocytes (details in Methods section), along with antigen-presenting cells. In the tetramer stimulation, tetramer and anti-CD28 were used as the molecular stimulation. Values plotted are mean ± s.e.m, with a statistical comparison between experimental conditions provided in the inset table (* P < 0.05, ** P < 0.005). (DOCX) [file pone.0191634.s012.docx]

**
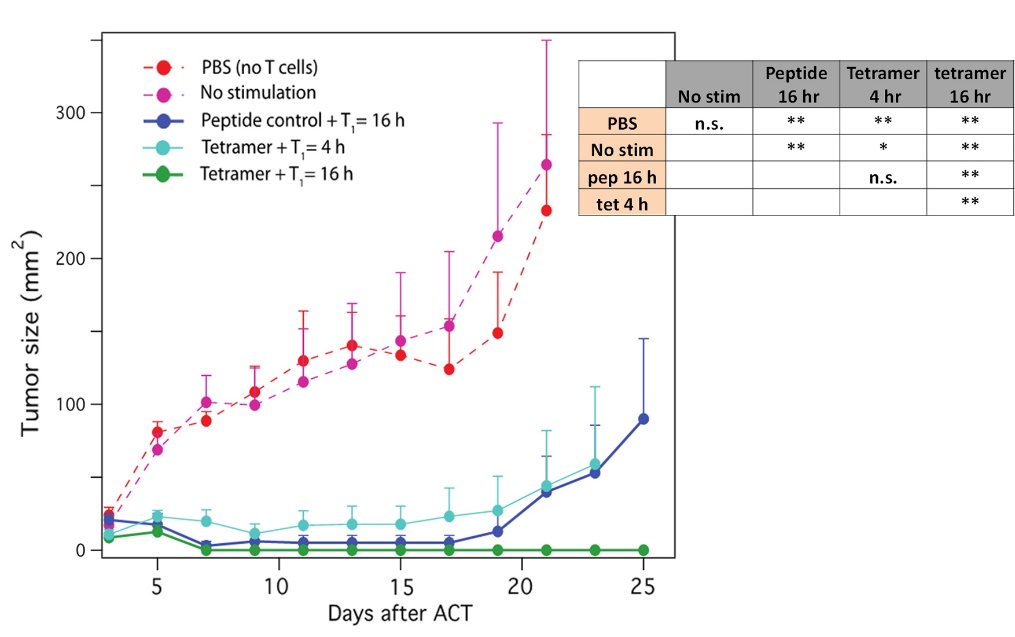
**

**S7 Fig. *In vivo* antitumor efficacy with peptide control vs. selected conditions in Figure 2.** For the peptide control, OVA peptide and IL2 were added directly to the splenocytes (details in methods section), along with antigen-presenting cells. In the tetramer stimulation, tetramer and anti-CD28 were used as the molecular stimulation. Values plotted are mean ± s.e.m, with a statistical comparison between experimental conditions provided in the inset table (*** *P* < 0.05, ** *P* < 0.005).
