# Supplementary material for: A kinetic investigation of interacting, stimulated T cells identifies conditions for rapid functional enhancement, minimal phenotype differentiation, and improved adoptive cell transfer tumor eradication
Source: PLoS One. 2018 Jan 23;13(1):e0191634. doi: 10.1371/journal.pone.0191634 (PMC5779691; doi:10.1371/journal.pone.0191634)
Supplement: S8 Fig — Hematoxylin staining demonstrates increased number of apoptotic cells that are shrunken with pyknotic and fragmented nuclei and condense cytoplasm after adoptive transfer of CD8+ T cells under 16-hour T1 conditioning with Ova tetramer and anti-CD28 stimulation (A) compared to non-stimulated CD8+ T cells (B) and without adoptive T cell transfer (C). Representative hematoxylin-stained sections are displayed. Bar, 20 μm. (DOCX) [file pone.0191634.s013.docx]

**S8 Fig. Gross cell morphology of EG.7 tumor 4 days after ACT under various conditions.** Hematoxylin staining demonstrates increased number of apoptotic cells that are shrunken with pyknotic and fragmented nuclei and condense cytoplasm after adoptive transfer of CD8^+^ T cells under 16-hour T_1_ conditioning with Ova tetramer and anti-CD28 stimulation (A) compared to non-stimulated CD8^+^ T cells (B) and without adoptive T cell transfer (C). Representative hematoxylin-stained sections are displayed. Bar, 20 μm.
