# Supplementary material for: A kinetic investigation of interacting, stimulated T cells identifies conditions for rapid functional enhancement, minimal phenotype differentiation, and improved adoptive cell transfer tumor eradication
Source: PLoS One. 2018 Jan 23;13(1):e0191634. doi: 10.1371/journal.pone.0191634 (PMC5779691; doi:10.1371/journal.pone.0191634)
Supplement: S9 Fig — Ki67 staining demonstrates decreased numbers of proliferating cells after adoptive transfer of CD8+ T cells under 16-hour T1 conditioning with Ova tetramer and anti-CD28 stimulation (A) compared to non-stimulated CD8+ T cells (B) and without adoptive T cell transfer (C). Representative examples are shown. Bar, 50 μm. (D) The proliferation index (mean ± s.e.m) was quantified using an intensity threshold. Analysis was done by one-way ANOVA followed by Bonferroni’s multiple comparison test (*:p < 0.05). The quantification was based on 4 fields per section (n = 4–8 histological sections per animal; 3–4 animals per group). (DOCX) [file pone.0191634.s014.docx]

**S9 Fig. The level of proliferation in EG.7 tumor after 4 days after ACT under various conditions.** Ki67 staining demonstrates decreased numbers of proliferating cells after adoptive transfer of CD8+ T cells under 16-hour T_1_ conditioning with Ova tetramer and anti-CD28 stimulation (A) compared to non-stimulated CD8+ T cells (B) and without adoptive T cell transfer (C). Representative examples are shown. Bar, 50 μm. (D) The proliferation index (mean ± s.e.m) was quantified using an intensity threshold. Analysis was done by one-way ANOVA followed by Bonferroni's multiple comparison test (*:p < 0.05). The quantification was based on 4 fields per section (*n* = 4-8 histological sections per animal; 3-4 animals per group).
