# Supplementary material for: A kinetic investigation of interacting, stimulated T cells identifies conditions for rapid functional enhancement, minimal phenotype differentiation, and improved adoptive cell transfer tumor eradication
Source: PLoS One. 2018 Jan 23;13(1):e0191634. doi: 10.1371/journal.pone.0191634 (PMC5779691; doi:10.1371/journal.pone.0191634)
Supplement: S10 Fig — The total area (means ± s.e.m) was quantified using an intensity threshold. Analysis was done by one-way ANOVA followed by Bonferroni’s multiple comparison test (**: P < 0.01). The quantification was based on 4 fields per section (n = 4–6 histological sections per animal; 4–5 animals per group). (DOCX) [file pone.0191634.s015.docx]

**S10 Fig**. **Quantification of CD137 staining.** The total area (means ± s.e.m) was quantified using an intensity threshold. Analysis was done by one-way ANOVA followed by Bonferroni's multiple comparison test (**: *P* < 0.01). The quantification was based on 4 fields per section (*n* = 4-6 histological sections per animal; 4-5 animals per group).
