# Supplementary material for: A kinetic investigation of interacting, stimulated T cells identifies conditions for rapid functional enhancement, minimal phenotype differentiation, and improved adoptive cell transfer tumor eradication
Source: PLoS One. 2018 Jan 23;13(1):e0191634. doi: 10.1371/journal.pone.0191634 (PMC5779691; doi:10.1371/journal.pone.0191634)
Supplement: S11 Fig — Flow cytometric determination of OT1+ T cells in mouse tumor biopsy 4 days after ACT of OT1 T cells with 16-hour T1 conditioning with molecular stimulation (OT1 tetramer + anti-CD28), or no stimulation. (DOCX) [file pone.0191634.s016.docx]

**
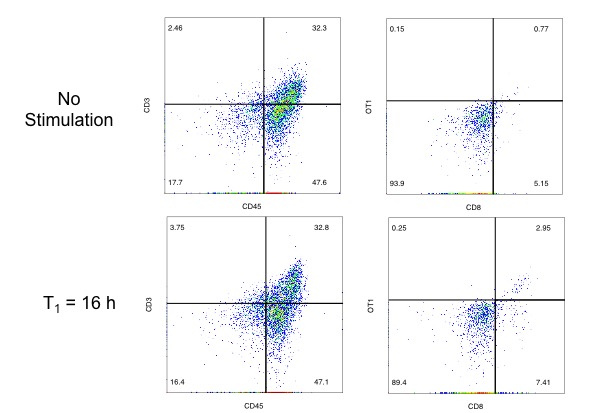
**

**S11 Fig. Population of OT1+ cells that infiltrate into the tumor site.** Flow cytometric determination of OT1+ T cells in mouse tumor biopsy 4 days after ACT of OT1 T cells with 16-hour T_1_ conditioning with molecular stimulation (OT1 tetramer + anti-CD28), or no stimulation.
