# Supplementary material for: A kinetic investigation of interacting, stimulated T cells identifies conditions for rapid functional enhancement, minimal phenotype differentiation, and improved adoptive cell transfer tumor eradication
Source: PLoS One. 2018 Jan 23;13(1):e0191634. doi: 10.1371/journal.pone.0191634 (PMC5779691; doi:10.1371/journal.pone.0191634)
Supplement: S12 Fig — (DOCX) [file pone.0191634.s017.docx]

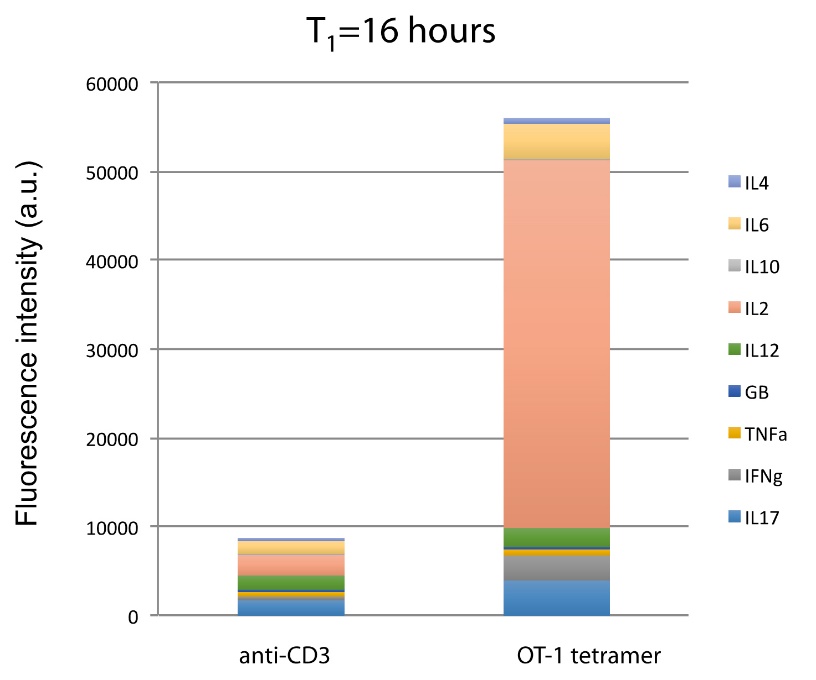


**S12 Fig. Ensemble cytokine secretion during T_1_=16 h for different molecular stimulation (anti-CD3 + anti-CD28, left) and (OT1 tetramer + anti-CD28).**
