# Supplementary material for: A kinetic investigation of interacting, stimulated T cells identifies conditions for rapid functional enhancement, minimal phenotype differentiation, and improved adoptive cell transfer tumor eradication
Source: PLoS One. 2018 Jan 23;13(1):e0191634. doi: 10.1371/journal.pone.0191634 (PMC5779691; doi:10.1371/journal.pone.0191634)
Supplement: S14 Fig — The secretion profile is largely similar for various T1 conditioning time. The absolute number of cells that secrete and the average secretion intensity, however, are significantly increased as T1 increases (shown in the main text). (DOCX) [file pone.0191634.s019.docx]

**S14 Fig. Relative number of cells that secrete a particular cytokine for human CD4^+^ T cells.** The secretion profile is largely similar for various T_1_ conditioning time. The absolute number of cells that secrete and the average secretion intensity, however, are significantly increased as T_1_ increases (shown in the main text).
