# Supplementary material for: A kinetic investigation of interacting, stimulated T cells identifies conditions for rapid functional enhancement, minimal phenotype differentiation, and improved adoptive cell transfer tumor eradication
Source: PLoS One. 2018 Jan 23;13(1):e0191634. doi: 10.1371/journal.pone.0191634 (PMC5779691; doi:10.1371/journal.pone.0191634)
Supplement: S15 Fig — A. Differential expressed genes relative to the 0-hour (NS) T1 conditioning are displayed with self-organizing map. B. Transcription factors that are enriched with the most dramatically up-regulated genes as T1 is increased from 0 hour (non-stimulated) to 2 hours. (DOCX) [file pone.0191634.s020.docx]

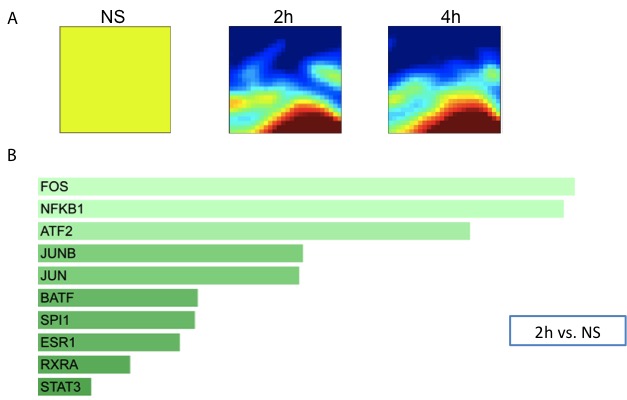


**S15 Fig. Enriched transcriptional network of human CD8^+^ T cells from transcriptome analysis.** A. Differential expressed genes relative to the 0-hour (NS) T_1_ conditioning are displayed with self-organizing map. B. Transcription factors that are enriched with the most dramatically up-regulated genes as T_1_ is increased from 0 hour (non-stimulated) to 2 hours.
