# Supplementary material for: A kinetic investigation of interacting, stimulated T cells identifies conditions for rapid functional enhancement, minimal phenotype differentiation, and improved adoptive cell transfer tumor eradication
Source: PLoS One. 2018 Jan 23;13(1):e0191634. doi: 10.1371/journal.pone.0191634 (PMC5779691; doi:10.1371/journal.pone.0191634)
Supplement: S19 Fig — (DOCX) [file pone.0191634.s024.docx]

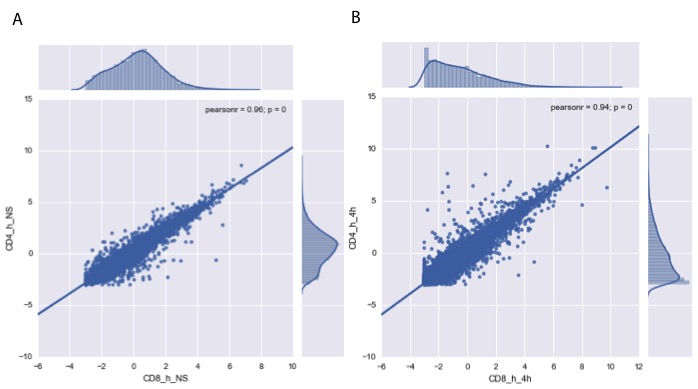


**S19 Fig. Correlation of human (A) CD4^+^ and (B) CD8^+^ T cells from the same patient as T_1_ increases from 0 hour (NS) to 4 hours.**
