# Supplementary material for: A kinetic investigation of interacting, stimulated T cells identifies conditions for rapid functional enhancement, minimal phenotype differentiation, and improved adoptive cell transfer tumor eradication
Source: PLoS One. 2018 Jan 23;13(1):e0191634. doi: 10.1371/journal.pone.0191634 (PMC5779691; doi:10.1371/journal.pone.0191634)
Supplement: S20 Fig — A. Phenotype analysis from multi-color flow cytometry of CD4+ T cells shows loss of the naïve phenotype, but no evidence of terminal differentiation. B. The expression level of naïve-associated (upper) and effector-associated (lower) genes as a function of T1 for CD4+ T cells. (DOCX) [file pone.0191634.s025.docx]

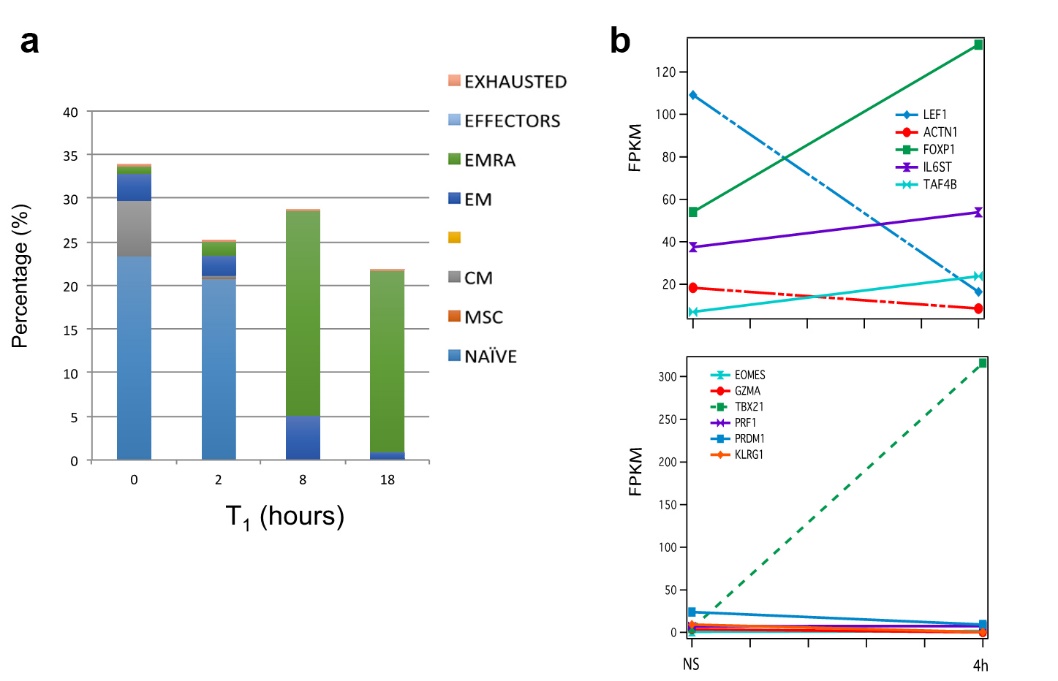


**S20 Fig. Phenotypic evolution and transcriptome dynamics for human CD4^+^ T cells after various T_1_ conditioning time.** A. Phenotype analysis from multi-color flow cytometry of CD4^+^ T cells shows loss of the naïve phenotype, but no evidence of terminal differentiation. B.The expression level of naïve-associated (upper) and effector-associated (lower) genes as a function of T_1_ for CD4^+^ T cells.
