# Supplementary material for: A kinetic investigation of interacting, stimulated T cells identifies conditions for rapid functional enhancement, minimal phenotype differentiation, and improved adoptive cell transfer tumor eradication
Source: PLoS One. 2018 Jan 23;13(1):e0191634. doi: 10.1371/journal.pone.0191634 (PMC5779691; doi:10.1371/journal.pone.0191634)
Supplement: S21 Fig — A. Characterization of secreted cytokines (CCL3 (upper) and IL2 (lower)) for OT1 T cells with molecular stimulation (OT1 tetramer + anti-CD28 + PMA + Ionomycin) under different cell densities (higher: 5 × 10^5^/ml, lower: 2 × 10^5^/ml) as T1 increases. B. Calibration curve of fluorescence signal vs. protein concentration. (DOCX) [file pone.0191634.s026.docx]

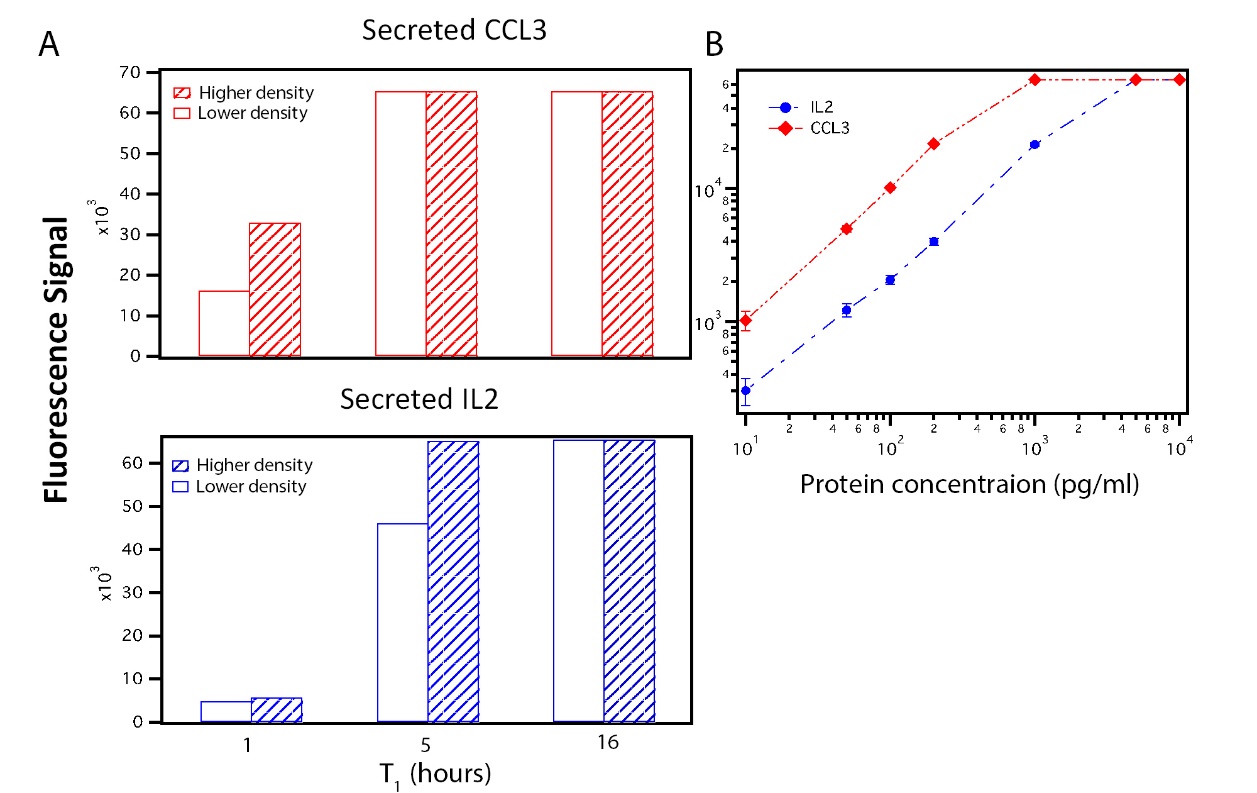


**S21 Fig.** **Cytokine secretion dynamics and calibration curve**. A. Characterization of secreted cytokines (CCL3 (upper) and IL2 (lower)) for OT1 T cells with molecular stimulation (OT1 tetramer + anti-CD28 + PMA + Ionomycin) under different cell densities (higher: 5 × 10^5^/ml, lower: 2 × 10^5^/ml) as T_1_ increases. B. Calibration curve of fluorescence signal vs. protein concentration.
