# Supplementary material for: A kinetic investigation of interacting, stimulated T cells identifies conditions for rapid functional enhancement, minimal phenotype differentiation, and improved adoptive cell transfer tumor eradication
Source: PLoS One. 2018 Jan 23;13(1):e0191634. doi: 10.1371/journal.pone.0191634 (PMC5779691; doi:10.1371/journal.pone.0191634)
Supplement: S22 Fig — Representative images of the clustering area under different cell densities (upper: 2 × 10^5^/ml, lower: 5 × 10^5^/ml). Scale bar = 200 μm. (DOCX) [file pone.0191634.s027.docx]

**S22 Fig.** **Cell clustering dynamics with strong molecular stimulation** (OT1 tetramer + anti-CD28 + PMA + ionomycin)**.** Representative images of the clustering area under different cell densities (upper: 2 × 10^5^/ml, lower: 5 × 10^5^/ml). Scale bar = 200 μm.
