# Supplementary material for: A kinetic investigation of interacting, stimulated T cells identifies conditions for rapid functional enhancement, minimal phenotype differentiation, and improved adoptive cell transfer tumor eradication
Source: PLoS One. 2018 Jan 23;13(1):e0191634. doi: 10.1371/journal.pone.0191634 (PMC5779691; doi:10.1371/journal.pone.0191634)
Supplement: S23 Fig — A consistent elevation was observed for OT1 CD8+ T cells, human CD4+ and CD8+ T cells. (DOCX) [file pone.0191634.s028.docx]

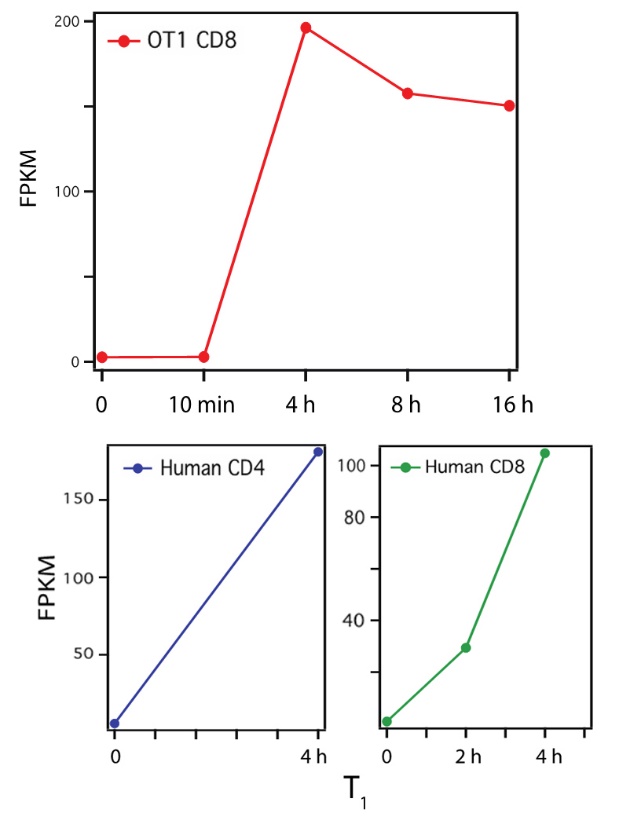


**S23 Fig. The RNA expression level of IL2 receptor (IL2RA) as a function of T_1_ conditioning time.** A consistent elevation was observed for OT1 CD8^+^ T cells, human CD4^+^ and CD8^+^ T cells.
