# Supplementary material for: A kinetic investigation of interacting, stimulated T cells identifies conditions for rapid functional enhancement, minimal phenotype differentiation, and improved adoptive cell transfer tumor eradication
Source: PLoS One. 2018 Jan 23;13(1):e0191634. doi: 10.1371/journal.pone.0191634 (PMC5779691; doi:10.1371/journal.pone.0191634)
Supplement: S1 Table — (DOCX) [file pone.0191634.s029.docx]

**S1 Table. List of antibody panel used for flow cytometry immuno-phenotyping analysis for human patient sample.**

| **Fluorochrome** | **Panel 1** | **Panel 2** | **Panel 3** |
| --- | --- | --- | --- |
| BV421 | CD45RA  (HI100✪) | CD154  (24-31✪) | CD69  (FN50✪) |
| BV510 | CD4  (OKT-4✪) | CD4  (OKT-4✪) | CD4  (OKT-4✪) |
| BV 605 | CD8  (RPA-T8✪) | CD8  (RPA-T8✪) | CD8  (RPA-T8✪) |
| BV 650 | CD3  (OKT-3✪) | CD3  (OKT-3✪) | CD3  (OKT-3✪) |
| FITC | CD57  (NK1★) | CD28  (CD28.2★) | **-** |
| PE | CCR7  (3D12★) | CD122  (Mikβ-3★) | CTLA4  (L3D10✪) |
| 7AAD | 7AAD◼ | 7AAD◼ | 7AAD◼ |
| ECD | CD62L  (DRG56◼) | HLA-DR  (Immu357◼) | **-** |
| APC/AlexaFluor647 | PD1  (MIH4*) | CD137  (4B4-1◼) | TIM3  (F38-2E2*) |
| AlexaFluor700 | CD27  (M-T271★) | **-** | **-** |
| APC-Cy7/APC-eF780 | CD25  (M-A251★) | CD40  (5C3**) | **-** |
| PE-Cy7 | CD95  (DX2★) | ICOS  (C389.4A✪) | **-** |
| BV711 | CD45RO  (UCHL1✪) | **-** | **-** |
| BV785 | CD127  (A019D5✪) | **-** | **-** |

Biolegend; *Affimetrix (eBioscience); ◼Beckman Coulter; ★BDbioscience. BV=Brilliant Violet; Ax=Alexa Fluor; ECD=PE-TexasRed; FTIC= Fluorescein isothiocyanate; PE= Phycoerythrin; APC= Allophycocyanin. PC5=Phycoerythrin Cyanine 5
