# Supplementary material for: A kinetic investigation of interacting, stimulated T cells identifies conditions for rapid functional enhancement, minimal phenotype differentiation, and improved adoptive cell transfer tumor eradication
Source: PLoS One. 2018 Jan 23;13(1):e0191634. doi: 10.1371/journal.pone.0191634 (PMC5779691; doi:10.1371/journal.pone.0191634)
Supplement: S2 Table — (DOCX) [file pone.0191634.s030.docx]

**S2 Table. List of antibody panel for human T cells in Single Cell Barcode Chip (SCBC).**

| Antibody | Manufacturer |
| --- | --- |
| IL-2 | R&D |
| IL-17 | R&D |
| IFN-γ | R&D |
| TNF-α | Biolegend |
| CCL4 | R&D |
| Perforin | Mabtech |
| Granzyme B | R&D |
| IL-4 | Biolegend |
| IL-6 | R&D |
| IL-10 | R&D |
| TGF-β | R&D |
