# Supplementary material for: A kinetic investigation of interacting, stimulated T cells identifies conditions for rapid functional enhancement, minimal phenotype differentiation, and improved adoptive cell transfer tumor eradication
Source: PLoS One. 2018 Jan 23;13(1):e0191634. doi: 10.1371/journal.pone.0191634 (PMC5779691; doi:10.1371/journal.pone.0191634)
Supplement: S3 Table — (DOCX) [file pone.0191634.s031.docx]

**S3 Table. List of antibody panel for mouse T cells in SCBC**

| Antibody | Manufacturer |
| --- | --- |
| IL-2 | R&D |
| IL-17 | R&D |
| IFN-γ | R&D |
| TNF-α | Biolegend |
| CCL3 | R&D |
| Perforin | Mabtech |
| Granzyme B | R&D |
| IL-4 | Biolegend |
| IL-6 | R&D |
| IL-10 | R&D |
| IL-12 | Biolegend |
